# TAM mediates adaptation of carbapenem-resistant *Klebsiella pneumoniae* to antimicrobial stress during host colonization and infection

**DOI:** 10.1371/journal.ppat.1009309

**Published:** 2021-02-08

**Authors:** Hea-Jin Jung, Matthew T. Sorbara, Eric G. Pamer

**Affiliations:** 1 Duchossois Family Institute, The University of Chicago, Chicago, Illinois, United States of America; 2 Department of Microbiology, The University of Chicago, Chicago, Illinois, United States of America; 3 Department of Medicine, Section of Infectious Diseases and Global Health, The University of Chicago, Chicago, Illinois, United States of America; Tufts University, UNITED STATES

## Abstract

Gram-negative pathogens, such as *Klebsiella pneumoniae*, remodel their outer membrane (OM) in response to stress to maintain its integrity as an effective barrier and thus to promote their survival in the host. The emergence of carbapenem-resistant *K*. *pneumoniae* (CR-*Kp*) strains that are resistant to *virtually* all antibiotics is an increasing clinical problem and OM impermeability has limited development of antimicrobial agents because higher molecular weight antibiotics cannot access sites of activity. Here, we demonstrate that TAM (translocation and assembly module) deletion increases CR*-Kp* OM permeability under stress conditions and enhances sensitivity to high-molecular weight antimicrobials. SILAC-based proteomic analyses revealed mis-localization of membrane proteins in the TAM deficient strain. Stress-induced sensitization enhances clearance of TAM-deficient CR*-Kp* from the gut lumen following fecal microbiota transplantation and from infection sites following pulmonary or systemic infection. Our study suggests that TAM, as a regulator of OM permeability, represents a potential target for development of agents that enhance the effectiveness of existing antibiotics.

## Author summary

Antibiotics remain remarkably effective at controlling bacterial infections, however, in part due to their extensive use in clinical and agricultural settings, many microbial pathogens have developed antibiotic resistance. Development of new antimicrobial agents to treat multidrug-resistant infections is particularly challenging for Gram-negative bacteria such as *Klebsiella pneumoniae*, a leading cause of nosocomial infection. Those bacteria have an additional layer in the envelope, which prevents potentially clinically useful antimicrobial compounds to access sites of activity. In this study, we demonstrate that translocation and assembly module (TAM) mediates antibiotic and antimicrobial peptide resistance during host colonization and infection with highly antibiotic-resistant *K*. *pneumoniae* (CR*-Kp*). Loss of TAM impaired the envelope biogenesis and increased the sensitivity of CR*-Kp* to large antimicrobials under stress conditions. Stress-induced sensitization enhanced clearance of CR*-Kp* from the gut lumen as well as from infection sites. Our finding offers a potential target for therapeutic agents to enhance permeability and thus effectiveness of existing and potential antibiotics.

## Introduction

Many microbial pathogens have developed antibiotic resistance [1], and alternative or even novel therapies may be required to treat or prevent infections. However, development of new antimicrobial agents to clear multidrug-resistant pathogens is particularly challenging for Gram-negative bacteria such as *Klebsiella pneumoniae*, a leading cause of nosocomial infection including pneumonia, urinary tract infection, bacteremia, and liver abscesses [2]. In contrast to Gram-positive bacteria, which are enveloped by a cytoplasmic membrane (inner membrane; IM) and a peptidoglycan cell wall, Gram-negative bacteria have an additional outer membrane (OM) that is highly impermeable and functions as an effective barrier against many extracellular molecules, including potentially clinically useful antimicrobial compounds [3]. Thus, Gram-negative bacteria are intrinsically resistant to large antibiotics that cannot pass through the OM [4–6]. Pathogenic and commensal bacteria have been shown to modify components of the OM to increase resistance to antimicrobials [4–7].

To overcome this barrier, substantial efforts have been made to design antimicrobial compounds with enhanced OM permeability [6]. Inhibition of OM biogenesis is a potential strategy since disruption of the OM, by interfering with solute traffic, is bactericidal but it can also synergize with other antibiotics by enhancing permeability across the OM [5,6,8]. In that context, chemical compounds that inhibit OM assembly have been explored—ACHN-975 (Achaogen) and POL7080/Murepavadin (Polyphor AG) target the LPS biosynthesis pathway [6]; MAC13243 and JB-95 target the Lol (lipoprotein outer membrane localization) and BAM (β-barrel assembly machinery) pathways, respectively [8]. When OM assembly is impaired, the void is filled by phospholipids with a higher permeability [4]. The transport system that mediates the translocation of phospholipids between the IM and the OM can be another target, but its molecular mechanism remains largely undetermined [8–14].

In a recent study, we identified bacterial factors that enable high-density persistence of carbapenem-resistant *K*. *pneumoniae* (CR*-Kp*) in the gut lumen of antibiotic-treated mice [15]. Among the isogenic mutants tested for fitness, Δ*tamA* showed the most dramatic defect, resulting in 4–5 log10 loss in 7 days when competed with a wild type strain. *tamA* encodes the outer membrane component of the translocation and assembly module (TAM) [16]. Recent studies suggested that TAM, together with the BAM complex, plays important roles in the assembly of outer membrane proteins, including various types of fimbriae [16–19]. While TAM has a distinct structure from the BAM complex, its functional distinction and substrate specificity remain unclear [20,21].

Here, we investigated how TAM contributes not only to gut colonization but also to pulmonary and systemic infection by ST258 CR*-Kp*. Loss of TAM increased the sensitivity of CR*-Kp* to vancomycin, a large OM-impermeable antibiotic, and also to an antimicrobial peptide, nisin, under stress conditions. Increased sensitivity resulted from stress-induced increase of OM permeability, which enhanced clearance of CR*-Kp* from the densely colonized gut by fecal microbiota transplantation (FMT) or transfer of bacterial consortia. The absence of TAM also rendered CR*-Kp* more susceptible to clearance by the innate immune system during lung infection and bacteremia.

## Results

### Growth of Δ*tamA* is impaired in cecal filtrates from antibiotic-treated mice under low osmotic stress

**Fig 1:**
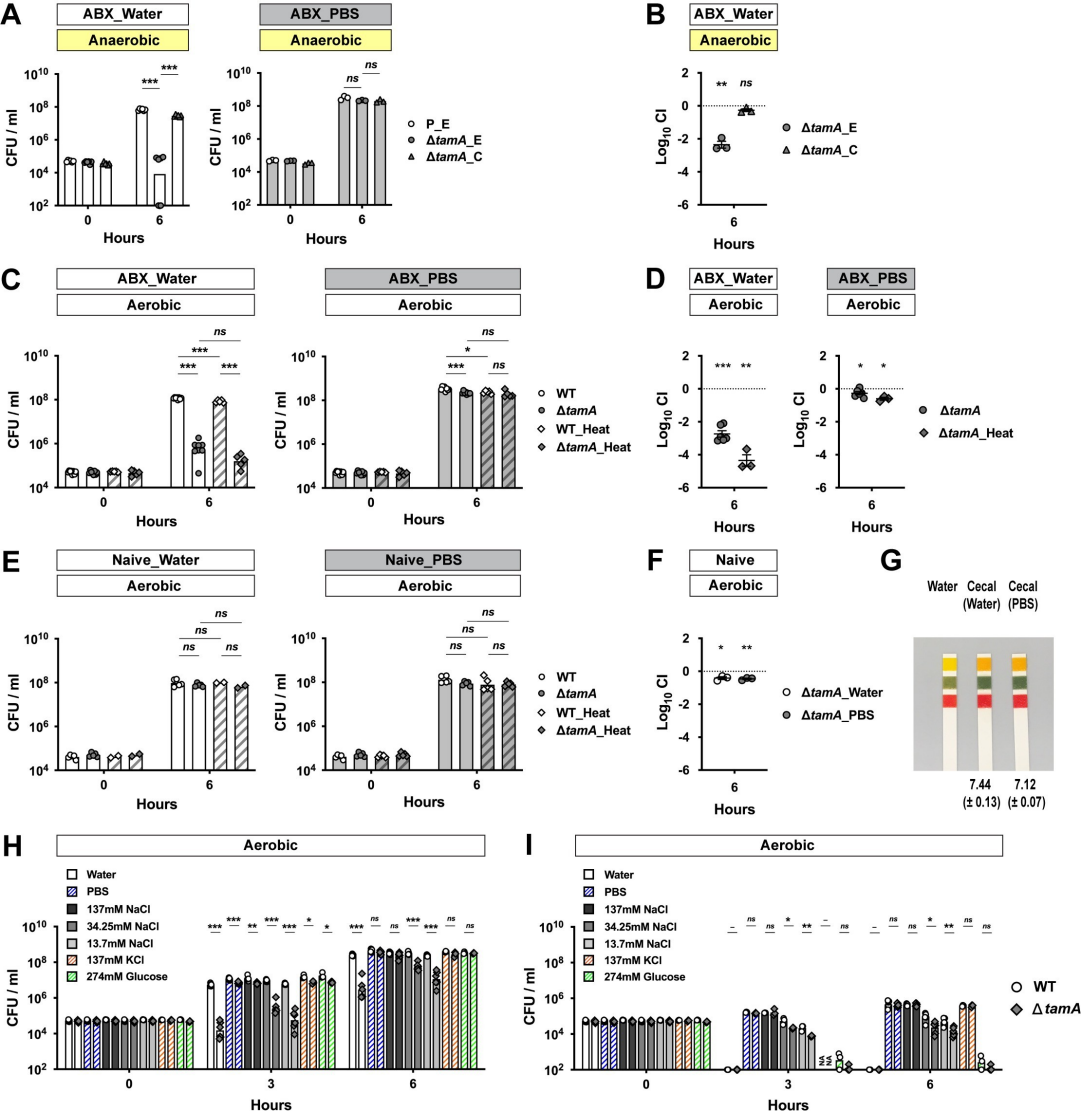
*ΔtamA* growth is reduced in cecal filtrates from antibiotic-treated mice under low osmotic stress. (A) A wild type strain harboring an empty pACYC177_aadA plasmid (P_E) and Δ*tamA* harboring either an empty pACYC177_aadA plasmid (Δ*tamA*_E) or a complementary plasmid, pTam, (Δ*tamA*_C) were mono-cultured anaerobically in either water- or PBS-suspended cecal filtrates from antibiotic-treated mice. In the water-suspended cecal filtrate, the growth of Δ*tamA*_E was significantly delayed compared to wild type while the growth defect was corrected in Δ*tamA*_C. In contrast, growth of all three strains was comparable in the PBS-suspended cecal filtrate. (B) Wild type (P_E) and one of the Δ*tamA* strains (Δ*tamA*_E or Δ*tamA*_C) were co-cultured in water-suspended cecal filtrate from antibiotic-treated mice. (C, D) Aerobic growth of wild type and Δ*tamA* strains was compared in cecal filtrates from antibiotic-treated mice after heat inactivation by autoclaving: (C) mono-culture; (D) co-culture. (E, F) Wild type and Δ*tamA* strains were (E) mono-cultured or (F) co-cultured in water- or PBS-suspended cecal filtrates from naïve mice. (G) Water-suspended cecal filtrates from antibiotic-treated mice were slightly more basic than those suspended in PBS. pH of water-suspended cecal filtrates from antibiotic-treated mice (*n* = 4) was measured using a pH meter and mean pH ± SD values are noted. The shown pH paper image was taken for visualization from one of the experiments. (H, I) Wild type (white circles; the left of each bar graph pair) and Δ*tamA* (grey rhombuses; the right of each bar graph pair) strains were mono-cultured (H) in cecal filtrates from antibiotic–treated mice, suspended with water, PBS, 137 mM NaCl, 34.25 mM NaCl, 13.7 mM NaCl, 137 mM KCl, or 274 mM glucose, or (I) in the diluents (water, PBS, 137 mM NaCl, 34.25 mM NaCl, 13.7 mM NaCl, 137 mM KCl, or 274 mM glucose solutions). For each round of experiments, the same inoculum batch was used for (H) and (I). NA, data not available. (A, C, E, H, I) Bar graphs represent geometric means. (B, D, F) Mean ± SEM of log_10_CI (competitive index) is shown.–, *p* value not available; *ns*, not significant; *, *p* < 0.05; **, *p* < 0.01; ***, *p* < 0.001, by (A, C, E, H, I) unpaired multiple *t* test or (B, D, F) one-sample *t* test on log10 transformation. For a better sterility and feasibility, some experiments were performed in aerobic conditions (C–I).

Dense colonization of the gut is a complex process involving many microbial and host factors. To narrow down potential mechanisms underlying the colonization defect of Δ*tamA* [15], we compared the growth of wild type and Δ*tamA* in the cecal filtrates from antibiotic-treated mice (Fig 1). We hypothesized that if reduced gut colonization by Δ*tamA* results from defective nutrient utilization or resistance to antimicrobial molecules, as opposed to reduced adhesion, the growth of Δ*tamA* would be reduced in the cecal contents from antibiotic-treated mice. We suspended the cecal contents in either PBS or water. Although PBS is a physiological buffer, it does not preserve some properties of original cecal content, including pH, which can affect the growth of CR-*Kp* [22]. PBS can also chelate existing metal ions [23–25]. For these reasons, we tested both water and PBS. *K*. *pneumoniae* is a facultative anaerobe and the lower GI tract is mostly anaerobic, so we first performed *ex vivo* cecal culture experiments under anaerobic conditions (Fig 1A and 1B). When cecal contents from antibiotic-treated mice were suspended in water, the number of Δ*tamA* CFUs (colony-forming units) 6 h post inoculation was ~1000 fold lower than wild type; the growth defect was corrected with a complementing plasmid (Fig 1A and 1B). In contrast, growth of wild type and Δ*tamA* were comparable in cecal filtrates from antibiotic-treated mice suspended with PBS (Fig 1A). The growth defect of Δ*tamA* in water-suspended cecal filtrates from antibiotic-treated mice was also observed under aerobic conditions, and heat inactivation by autoclaving did not reduce the Δ*tamA* growth defect (Fig 1C and 1D). On the other hand, water or PBS suspension did not impact the growth of Δ*tamA* in cecal filtrates from naïve mice (Fig 1E and 1F). While CR-*Kp* can grow in fresh cecal filtrates, a previous study from our laboratory showed that growth of CR-*Kp* is suppressed in naïve cecal contents that have been cultured for 24 hours under anaerobic conditions, but not in antibiotic-treated cecal contents [22]. This apparent disparity is attributable to the high concentrations of SCFAs that accumulate in *ex vivo* cecal cultures (S1 Fig) [22].

The pH of water-suspended cecal filtrates from antibiotic-treated mice was slightly higher than that of PBS-suspension (Fig 1G) and thus does not explain the growth suppression of Δ*tamA*—the pH of cecal contents from naïve mice is in the range of pH5.5–6.0 and suppresses CR-*Kp* growth [22]. Therefore, we explored another possibility—we suspected that lower osmolality of water led to the differential phenotype of Δ*tamA* in water- and PBS-suspended cecal filtrates from antibiotic-treated mice. To test this idea, we suspended cecal contents from antibiotic-treated mice in sodium chloride solutions of different osmolalities (137mM NaCl for iso-osmolality and 34.25mM/13.7 mM NaCl for hypo-osmolality) and compared the growth of Δ*tamA* (Fig 1H). Indeed, the Δ*tamA* growth defect was observed when cecal contents were suspended in low salt solutions. In contrast, when the osmolality was adjusted with either potassium chloride or glucose, Δ*tamA* grew normally (Fig 1H). However, defective Δ*tamA* growth was not solely due to increased sensitivity to low osmotic stress—the growth defect was not observed in water-suspended cecal filtrates from naïve mice (Fig 1E). When wild type and Δ*tamA* were cultured in solutions that lacked cecal contents from antibiotic-treated mice, there was a statistically significant, but minor difference in survival (Fig 1I).

### Loss of TAM increases sensitivity of CR*-Kp* to large OM-impermeable antimicrobials under stress conditions

**Fig 2:**
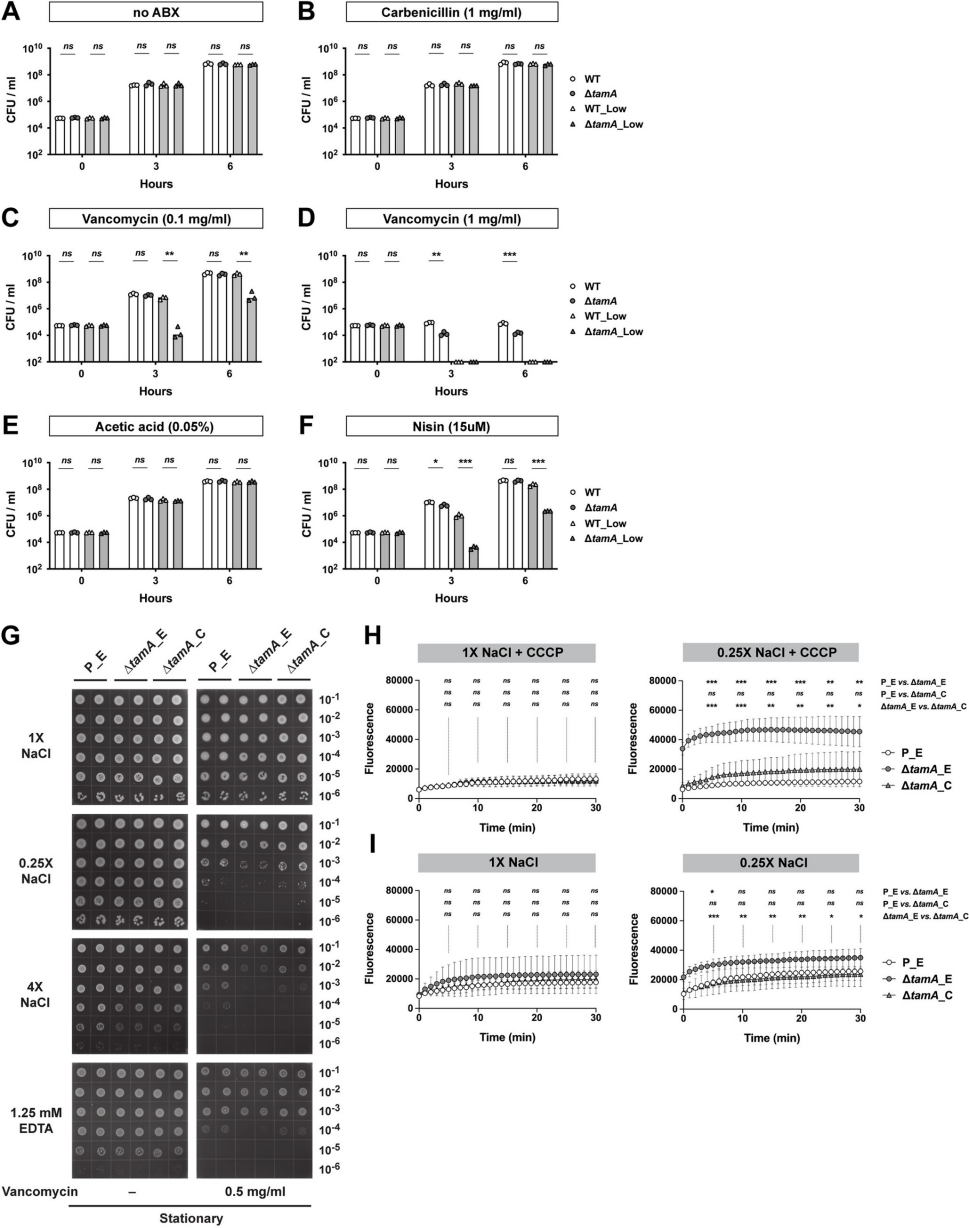
Loss of TAM increases sensitivity of CR*-Kp* to OM-impermeable antimicrobials under stress conditions, which results from increased permeability. (A–F) Wild type and Δ*tamA* strains were mono-cultured in regular (171mM NaCl; white bars) or low-salt (34mM NaCl; grey bars) LB media with (B) 1.0 mg/ml of carbenicillin; (C) 0.1 mg/ml or (D) 1.0 mg/ml of vancomycin; (F) 2.0 mg/ml (~15uM) of nisin. Growth in LB media with acetic acid, a solvent for nisin, is shown in (E). The same inoculum batches were used for (A–D) and for (E, F). Bar graphs represent geometric means. *ns*, not significant; *, *p* < 0.05; **, *p* < 0.01; ***, *p* < 0.001, by unpaired multiple *t* test on log10 transformation. (G) 10-fold serial dilutions of stationary phase cultures were blotted on LB plates with different concentrations of NaCl or EDTA with or without 0.5 mg/ml of vancomycin. P_E, a wild type strain harboring an empty pACYC177_aadA plasmid; Δ*tamA*_E, Δ*tamA* harboring an empty pACYC177_aadA plasmid; Δ*tamA*_C, Δ*tamA* harboring a complementary plasmid, pTam. (See also S5 Fig). (H, I) Wild type and Δ*tamA* cells were suspended in isosmotic (with 137mM NaCl, 1X) or hypo-osmotic (with 34.25mM NaCl, 0.25X) buffers and analyzed for uptake of NPN in the (H) presence or (I) absence of CCCP. P_E, a wild type strain harboring an empty pACYC177_aadA plasmid; Δ*tamA*_E, Δ*tamA* harboring an empty pACYC177_aadA plasmid; Δ*tamA*_C, Δ*tamA* harboring a complementary plasmid, pTam. Means ± SD from 6 independent experiments are shown. *ns*, not significant; *, *p* < 0.05; **, *p* < 0.01; ***, *p* < 0.001, by two-way ANOVA tests for every 5 min.

While low osmolality contributes to the Δ*tamA* phenotype, Δ*tamA* did not show a gross growth defect in water-suspended naïve cecal filtrates (Fig 1E and 1F) or in the low salt solutions (Fig 1I). We also did not observe clear differences between wild type and Δ*tamA* in the use of diverse carbon sources or growth under osmotic and pH stresses. (S2 Fig). In contrast, Δ*tamA* CFUs in water-suspended cecal filtrate from antibiotic-treated mice at 3 h post inoculation was even lower than that of the inoculum (Fig 1H), implying active killing of Δ*tamA*, rather than slower growth. We speculated that Δ*tamA* might be more sensitive to certain antimicrobials and that low osmolality amplifies this phenotype. To explore this idea, we tested several antimicrobial compounds in a non-growing condition with PBS (S3 and S4 Figs). In PBS, none of the tested molecules discriminated wild type and Δ*tamA*—some showed statistically significant differences but they were minor in magnitude. However, under low osmotic pressure of ¼-diluted PBS, Δ*tamA* had increased susceptibility to hydrogen peroxide, triton X-100, and lithocholic acid (LCA) (S3B and S3D and S4B Figs); its sensitivity to SDS, polymyxin B, and other bile acids virtually did not differ from wild type. Interestingly, Δ*tamA* was also more susceptible to vancomycin, but not to metronidazole (S4D Fig)—two antibiotics that had been administered to mice to disrupt the normal gut flora to enable dense colonization with CR-*Kp* [15]. Gram-negative bacteria such as CR-*Kp* are intrinsically resistant to the glycopeptide antibiotic vancomycin [4–6] because it is too large to pass through outer membrane porins [26–28] and, since it is hydrophilic, it cannot freely diffuse across the OM. To investigate if the growth defect of Δ*tamA* relates to increased sensitivity to vancomycin, we compared the growth of wild type and Δ*tamA* in LB media with different concentrations of salts and antibiotics (Fig 2A–2D) and found that the Δ*tamA* phenotype was reproduced with low salt LB media supplemented with 0.1 mg/ml vancomycin (Fig 2C). A higher concentration of vancomycin (1 mg/ml) distinguished wild type and Δ*tamA* even in regular LB media (Fig 2D). When combined with low salt stress, 1 mg/ml vancomycin was too potent to allow even wild type CR-*Kp* to survive. The growth of wild type and Δ*tamA* was comparable in the media without any antibiotics (Fig 2A) and in the media with 1 mg/ml of carbenicillin (Fig 2B).

The selective sensitivity of Δ*tamA* to vancomycin, triton X-100, and LCA—but not to small antibiotics and rather water-soluble bile acids—and the further increase of the sensitivity under low osmotic pressure suggest that Δ*tamA* has a more permeable OM. To explore this idea, we tested susceptibility of Δ*tamA* to nisin [29,30], a natural antimicrobial peptide from *Lactococcus lactis*, to which Gram-negatives are generally resistant due to OM impermeability (Fig 2E and 2F)—0.05% acetic acid that was used to prepare nisin stock served as a control. Similar to vancomycin, Δ*tamA* showed higher sensitivity to nisin when cultured in low salt LB.

To determine whether the Δ*tamA* phenotype results from low osmotic stress or a more general cause of membrane dysfunction, we examined the impact of high salt and ethylenediaminetetraacetic acid (EDTA), a known membrane permeabilizer, on sensitivity to vancomycin (Figs 2G and S5). We used LB agar plates with different concentrations of NaCl/EDTA and vancomycin, and compared growth of wild type and Δ*tamA* strains carrying either an empty or a complementing plasmid. Consistent with the broth culture experiment (Fig 2C and 2D), Δ*tamA* showed higher sensitivity to vancomycin under low osmotic pressure while cells at stationary phase tended to be more resistant and required higher concentrations of vancomycin to be differentiated from wild type bacteria (Figs 2G and S5). The increased susceptibility of Δ*tamA* to vancomycin was corrected in the complemented strain. When Δ*tamA* was subjected to high salt stress, it also showed higher sensitivity to vancomycin, suggesting this effect is not purely attributable to low osmotic stress. Along similar lines, although the impact was relatively milder than the salt stress, EDTA increased Δ*tamA* sensitivity. This difference was more apparent when stationary phase cells were cultured with a higher amount of vancomycin (S5 Fig). Of note, both exponential and stationary phase cells re-enter exponential phase to form colonies on plates, so the differential sensitivity likely resulted from better survival of stationary phase cells during the early phase of growth resumption.

### The OM biogenesis is impaired in Δ*tamA*

The higher susceptibility of Δ*tamA* to OM-impermeable antimicrobials such as vancomycin and nisin suggests that the function of the OM as a permeability barrier is compromised. The augmenting impact of OM-disturbing stresses further supports this notion. To investigate more directly if the OM permeability is altered in Δ*tamA*, we compared the uptake of the fluorescent probe 1-*N*-phenylnaphthylamine (NPN) by wild type and Δ*tamA* (Fig 2H and 2I). NPN fluoresces in phospholipid environments—for example, when phospholipids in the inner leaflet of the OM become accessible as the OM is damaged—and has been used to examine OM barrier integrity [31,32]. To address the possibility of differential NPN efflux, we assessed development of NPN signals in the presence of carbonyl cyanide 3-chlorophenylhydrazone (CCCP), which de-energizes cells and prevents export of NPN (Fig 2H). In an isosmotic buffer (with 137mM NaCl), wild type and Δ*tamA* were indistinguishable. However, when tested in a hypo-osmotic buffer (with 34.25mM NaCl), the NPN signal was significantly higher in Δ*tamA*, indicating higher permeability of the OM. The leaky OM was corrected in the complemented strain. In the absence of CCCP, where the fluorescence signal resulted from net NPN influx and efflux, the gap between wild type and Δ*tamA* was reduced, suggesting that efflux pumps of Δ*tamA* function (Fig 2I).

**Fig 3:**
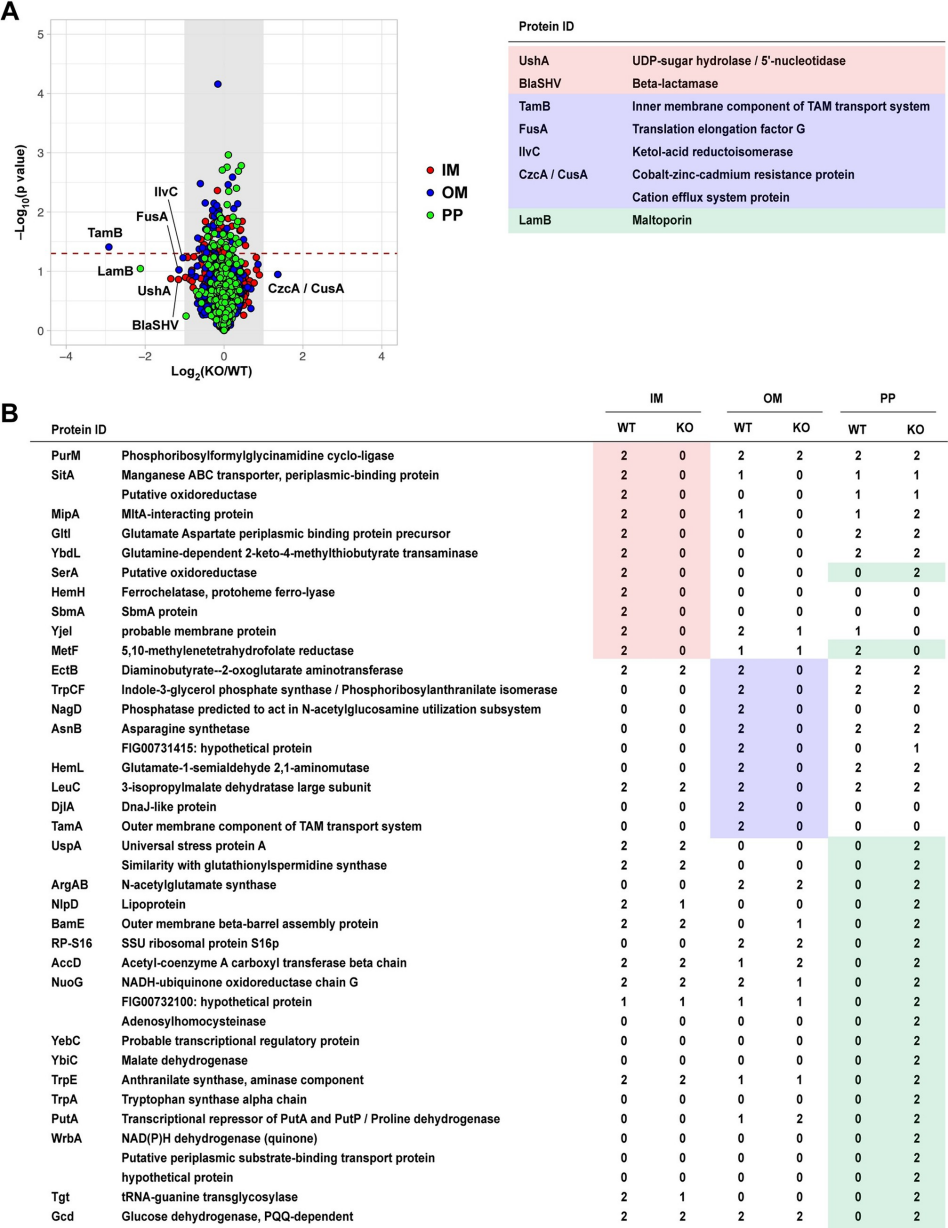
Abundance of a subset of proteins is altered in Δ*tamA* while overall protein profiles are conserved. (A) Protein profiles of inner membrane (IM), outer membrane (OM), and periplasmic (PP) fractions from wild type and Δ*tamA* strains were assessed by LC-MS/MS (see Materials and Methods for details), and average fold changes in proteins (Δ*tamA*/wild type) and *p*-values by one sample t-test are plotted in a log2 and log10 scales, respectively. The proteins detected in each sub-fraction are highlighted with colors: red, the IM fraction; blue, the OM fraction; green, the PP fraction. The grey shades mark the fold change cut-off of 2 and proteins that showed more than 2-fold changes in average are listed on the right. The dashed line indicates p-value of 0.05. Fold changes and *p*-values of all the plotted proteins can be found in S1 Table. (See also S7 Fig). Raw data of the LC-MS/MS analysis is available in S1 Data. (B) The list of proteins that were exclusively detected in either wild type or Δ*tamA*. The same color scheme to (A) is applied. The numbers (0–2) indicate the number of replicates in which the given protein is detected. For example, in the IM fraction, PurM was detected in wild type (WT) in both replicates (“2”) but not in Δ*tamA* (KO) in either replicate (“0”). The proteins listed here are not included in (A)—the fold changes cannot be calculated if proteins are not detected in either wild type or Δ*tamA* samples. (See also S2 Table).

Previous studies suggested that TAM enables integration of β-barrel proteins into the outer membrane [19]. To explore the possibility that the absence of the TAM leads to mis-localization of membrane proteins, we separated the IM, OM, PP sub-fractions of wild type and Δ*tamA* using selective detergents (S6 Fig) [33] and analyzed their protein profiles with LC-MS/MS (tandem mass spectrometry coupled with liquid chromatography) (Figs 3 and S7 and S1–S3 Tables). To minimize variation in sample handling during protein fractionation, we used SILAC (stable isotope labeling of amino acids in cell culture) with stable isotope–labeled lysine [34]. We detected more than 500 proteins in each fraction, with the majority present in similar amounts in both wild type and Δ*tamA* (Figs 3A and S7 and S1 Table). The levels of some proteins (*e*.*g*., UshA, FusA) differed in the range of 2–4 fold, but they did not reach statistical significance; TamB showed ~7.5 fold reduction in Δ*tamA* (*p* = 0.039) (Figs 3A and S7B and S1 Table). Although the overall protein profiles were not dramatically altered, several proteins were only detected in the sub-fractions of either wild type or Δ*tamA* (Fig 3B and S2 Table)—as expected, TamA was only found in the OM of wild type, but not in Δ*tamA*. Among those, proteins in the IM and OM fractions were exclusively found in wild type but not in Δ*tamA*. In contrast, many proteins including NlpD, a lipoprotein, and BamE, an outer membrane β-barrel assembly protein, were detected in the PP fraction of Δ*tamA*, but not in wild type. In the case of SerA, a putative oxidoreductase, it was found in the IM fraction for wild type, but in the PP fraction for Δ*tamA*. These findings support the idea that incorporation of membrane proteins is impaired in the absence of the TAM.

As the predicted function of the TAM is the assembly of β-barrel proteins including autotransporters [16], we further analyzed changes in the levels of β-barrel proteins, *in silico* predicted by BOMP [35] (S3A Table). Out of 35 proteins detected, excluding the TAM proteins, only two proteins were somewhat altered—MipA (peg #3747) was lost in Δ*tamA*; LamB (peg #5547) was ~2 fold lower in Δ*tamA* (*p* = 0.090) (Fig 3 and S3A Table). None of potential autotransporters (see Materials and Methods for details) showed significant changes in their abundance (S3B and S3C Table). Meanwhile, SDS-PAGE of WT and Δ*tamA* LPS grown under low-salt conditions revealed some changes, suggesting that TAM may alter LPS structure (S8B Fig). Gene expression of *tamA* and *tamB* were largely unaltered under osmotic stress (S9 Fig.).

### TAM deficiency enhances clearance of CR-*Kp* gut colonization by FMT

**Fig 4:**
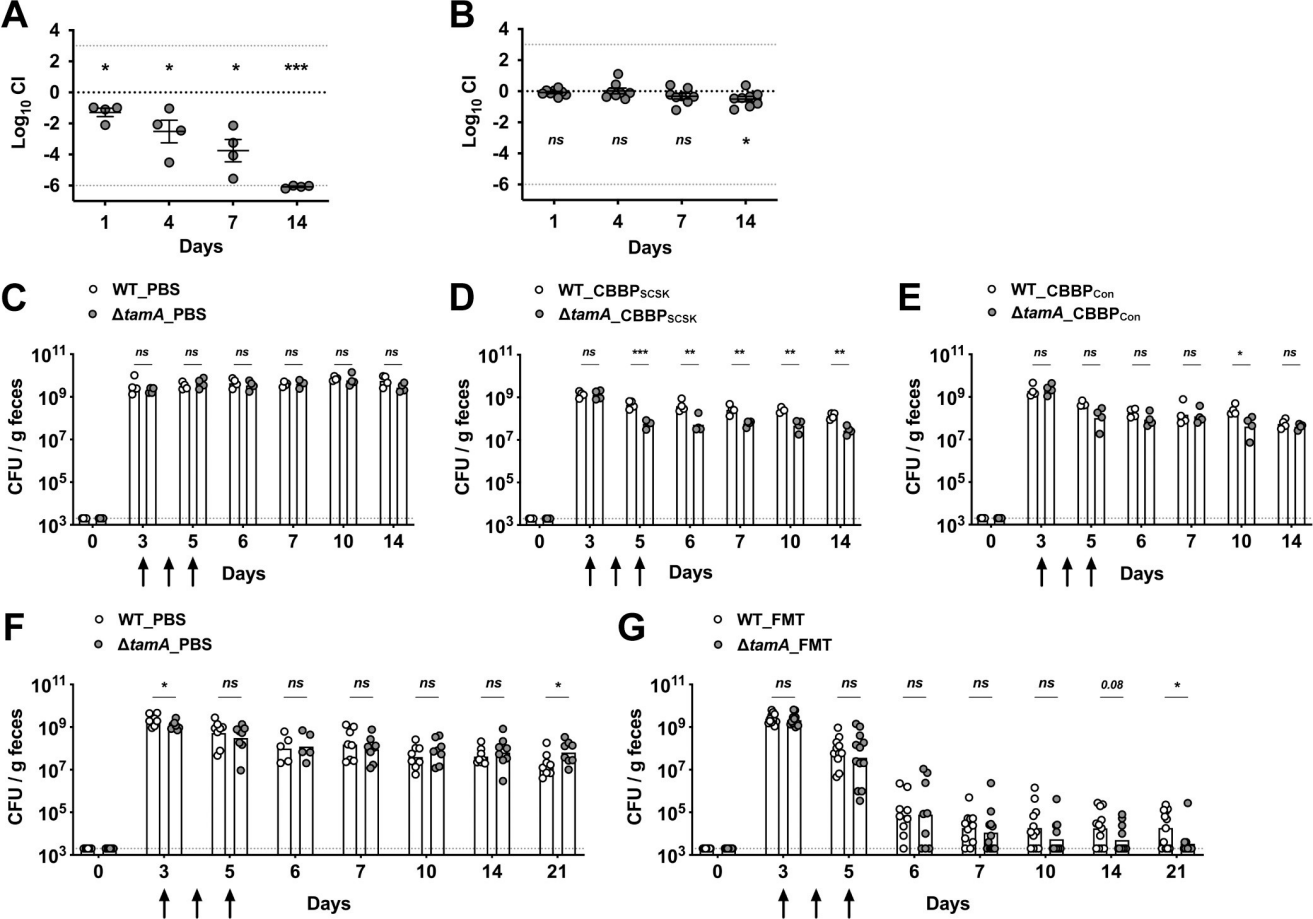
Δ*tamA* undergoes enhanced clearance from the gut following administration of CBBP or fecal microbiota transplantation (FMT). (A, B) Mice were treated with either (A) vancomycin or (B) ampicillin in drinking water and inoculated with 1:1 mixture of wild type and Δ*tamA* strains. The density of each strain in feces was determined over 14 days. Mean ± SEM of log_10_CI (competitive index) is shown. *ns*, not significant; *, *p* < 0.05; **, *p* < 0.01; ***, *p* < 0.001, by one-sample *t* test on log10 transformation of CI. (C–E) Ampicillin-treated mice were mono-colonized with wild type (open circles) or Δ*tamA* (closed circles) strains and clearance by PBS, CBBPSCSK [36,37], or CBBPCon was compared. The arrows indicate when PBS or either bacterial consortia were administered. Bar graphs represent geometric means and the dotted line indicates limit of detection. *ns*, not significant; *, *p* < 0.05; **, *p* < 0.01; ***, *p* < 0.001, by unpaired multiple *t* test on log10 transformation. (F, G) V+M (Vancomycin and metronidazole)–treated mice were mono-colonized with wild type or Δ*tamA* strains and clearance by PBS or FMT prepared from naïve mice was compared. The arrows indicate when PBS or FMT were administered, and bar graphs represent geometric means. *ns*, not significant; *, *p* < 0.05; **, *p* < 0.01; ***, *p* < 0.001, by unpaired multiple *t* test on log10 transformation.

To investigate the impact of the defective OM of Δ*tamA* on gut colonization, we performed competition assays in mice treated with different antibiotics (Fig 4A and 4B). Similar to our previous results using vancomycin and metronidazole [15], vancomycin alone depleted Δ*tamA* to the detection limit within 14 days (Fig 4A). In contrast, ampicillin administration did not result in reduced colonization by Δ*tamA* (Fig 4B), consistent with the *in vitro* studies (Fig 2B–2D).

Because Δ*tamA* has increased susceptibility to nisin (Fig 2F), we tested whether the four-strain consortium of commensal bacteria (CBBP_SCSK_)—containing a *Blautia producta*, BP_SCSK_, which produces a lantibiotic, similar to nisin-A [36,37]—can promote suppression of Δ*tamA* from the densely colonized gut (Fig 4C–4E). As expected, the consortium, initially assembled for the clearance of vancomycin-resistant *Enterococcus* [36,37], was not effective for the clearance of wild type (Fig 4D). However, while incomplete, the CBBP_SCSK_ consortium reduced the density of Δ*tamA* in the gut ~10 fold compared to wild type CR-*Kp*. When BP_SCSK_ was replaced by *B*. *producta* (Clostridiales VE202-06) that does not produce the lantibiotic, clearance of wild type and Δ*tamA* by the four-strain consortium (CBBP_Con_), was similar, with the exception of day 10 (Fig 4E).

We speculated that complex fecal materials from naïve mice would contain more diverse antimicrobials that Δ*tamA* is more sensitive to, leading to a clearer distinction between wild type and Δ*tamA*. Therefore, we compared clearance of wild type and Δ*tamA* from the densely colonized gut by fecal microbiota transplantation (FMT) (Fig 4F and 4G). In general, FMT was more effective than the four-strain consortium, lowering the CFU of wild type ~10^4^-folds. However, wild type persisted in the half of the mice about 2 weeks after the FMT at the density of ~10^5^ CFU /g feces. In contrast, Δ*tamA* was virtually undetectable in the majority of the mice by day 21. Of note, the CFU of Δ*tamA* in PBS control mice was a bit higher than wild type on day 21 (Fig 4F). This is likely due to differential recovery of background microbiota upon discontinuation of antibiotics.

### Δ*tamA* is cleared more rapidly by host defenses during lung infection and bacteremia

**Fig 5:**
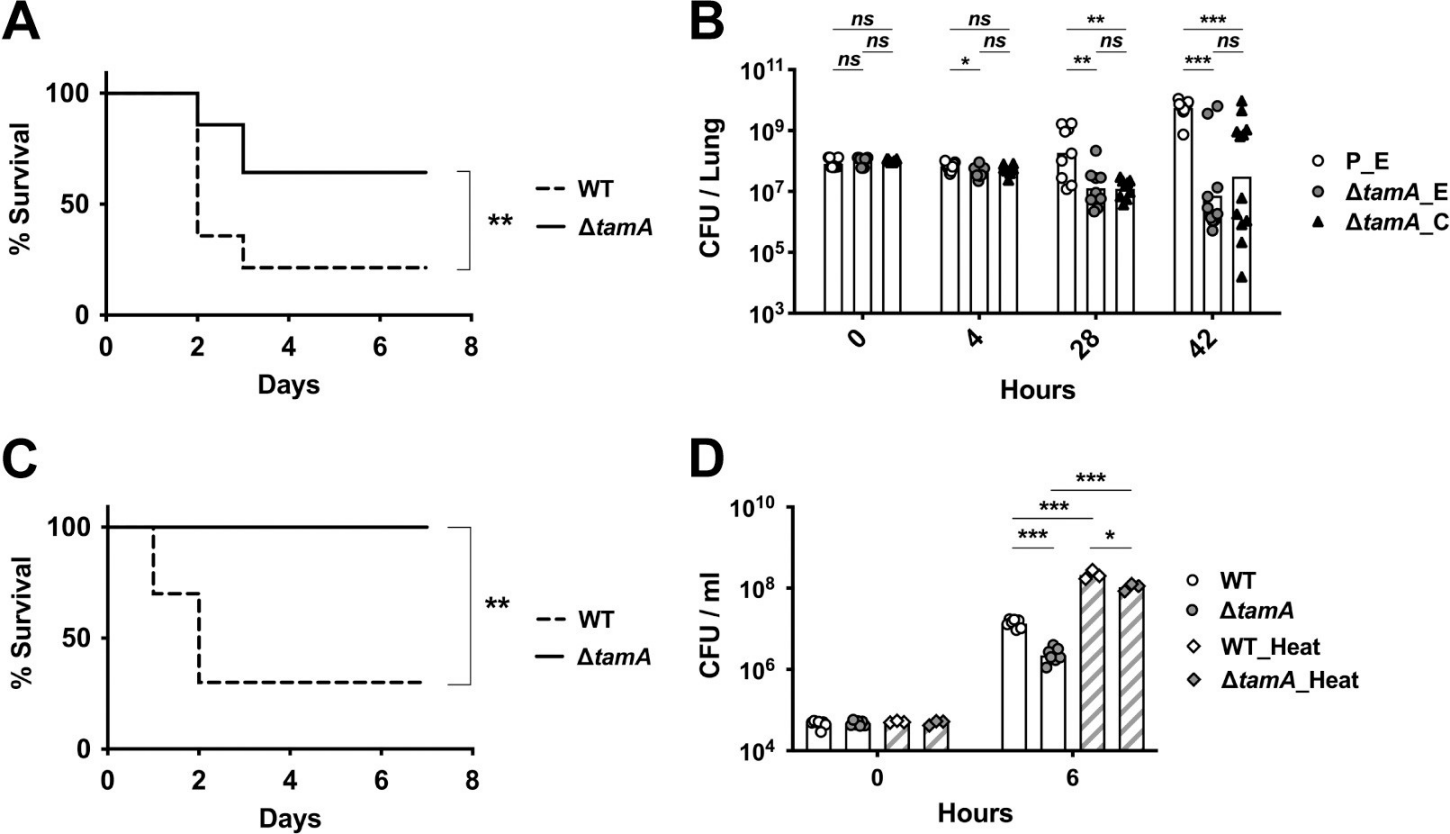
Δ*tamA* is more susceptible to clearance by host defenses during lung infection and bacteremia. (A) Mice were inoculated intratracheally with 10^8^ CFU of wild type or Δ*tamA* strains and mouse survival was monitored over 7 days (*n* = 14). **, *p* < 0.01 by Gehan-Breslow-Wilcoxon test. (B) The bacterial burden in lungs at 4, 28, 42 h after intratracheal inoculation (*n* = 12 for each group and time point). P_E, a wild type strain with an empty pACYC177_aadA plasmid; Δ*tamA*_E, Δ*tamA* with an empty pACYC177_aadA plasmid; Δ*tamA*_C, Δ*tamA* with a complementary plasmid, pTam. Bar graphs represent geometric means. *ns*, not significant; *, *p* < 0.05; **, *p* < 0.01; ***, *p* < 0.001, by unpaired multiple *t* test on log10 transformation. (C) Mice were inoculated intraperitoneally with 10^7^ CFU of wild type or Δ*tamA* strains and survival was monitored over 7 days (*n* = 10). **, *p* < 0.01 by Gehan-Breslow-Wilcoxon test. (D) Wild type and Δ*tamA* strains were mono-cultured in normal human sera without or with heat inactivation. Bar graphs represent geometric means. **, *p* < 0.01; ***, *p* < 0.001, by unpaired multiple *t* test on log10 transformation.

*Klebsiella pneumoniae* is an important cause of pulmonary infection. Given the increased sensitivity of Δ*tamA* to OM-impermeable antimicrobials, we hypothesized that its virulence may be reduced during pulmonary infection. To test this, we challenged naïve mice with wild type or Δ*tamA* strains by intratracheal inoculation (Fig 5A and 5B) and found 65% survival following pulmonary infection with Δ*tamA* versus 20% survival following wild type infection (Fig 5A). Increased survival of mice infected with Δ*tamA* was associated with enhanced clearance of Δ*tamA* from lung tissues (Fig 5B). During the early phase of infection (*i*.*e*., 4h post inoculation), the number of CFUs of wild type and Δ*tamA* in the lung were comparable. However, at later time points, the bacterial burden was significantly lower in the majority of the mice infected with Δ*tamA*. Enhanced clearance of Δ*tamA* was partially corrected in the complemented strain but did not reach statistical significance—possibly due to plasmid loss in the absence of *in vivo* selection (Fig 5B). Δ*tamA* also demonstrated reduced virulence during bacteremia, with 100% (Δ*tamA*) versus 30% (wild type) survival in 7 days following infection (Fig 5C). Lower virulence of Δ*tamA* during lung and bloodstream infection correlates with higher sensitivity to serum-mediated killing. When we compared the survival of wild type and Δ*tamA* in human serum, Δ*tamA* was more effectively killed than wild type, a difference that was partially lost by heat inactivation of serum (Fig 5D). Given the well-known function of the *K*. *pneumoniae* capsule in virulence and complement resistance [38], acapsular mutants (Δ*wza* and Δ*wzc*) [39] had reduced serum survival and led to less bacteremia than Δ*tamA* (S10 Fig).

## Discussion

To successfully colonize and infect hosts, bacterial pathogens need to cope with diverse physicochemical stresses, and the ability to resist these environmental stresses in part determines the pathogen’s susceptibility to host defenses and drives the evolution of bacterial pathogenesis mechanisms [40–42]. In our study, the Δ*tamA* phenotype was detectable *in vitro* only under OM-disturbing stresses. It was more apparent, however, in *in vivo* mouse models of pulmonary infection and bacteremia. The *in vivo* stresses that rendered Δ*tamA* more susceptible are likely multi-factorial [43] with tissue osmolality being contributory. As demonstrated by *in vitro* studies, osmotic stress sensitizes Δ*tamA* to OM-impermeable antimicrobials. In the gut lumen, osmolality can be much higher than the bloodstream, however it is easily and dramatically altered by food consumption, diarrhea or antibiotic treatment [40,41,44–46]. Osmotic stress responses may also contribute to reduced virulence of Δ*tamA* during infection as the osmolality of lung fluids can be also altered [47]. Antimicrobial peptides (AMPs) that are produced by the host, particularly Bactericidal/permeability increasing proteins (BPIs), also likely lead to sensitization. Similar to EDTA, AMPs destabilize LPS, damaging the outer membranes of Gram-negative bacteria [4,48–51]. A recent study suggested that serum complement can perturb the OM of Gram-negative bacteria, sensitizing them to antibiotics [52]. Consistent with this report, we demonstrated that Δ*tamA* is more susceptible to serum killing, a defect that was significantly corrected by heat inactivation of serum (Fig 5D). The low concentration of the OM-stabilizing divalent cations Mg^2+^ and Ca^2+^ in phagocytic vacuoles and osmotic stress in the host cells are additional potential contributors to the enhanced clearance of Δ*tamA* [4,53].

**Fig 6:**
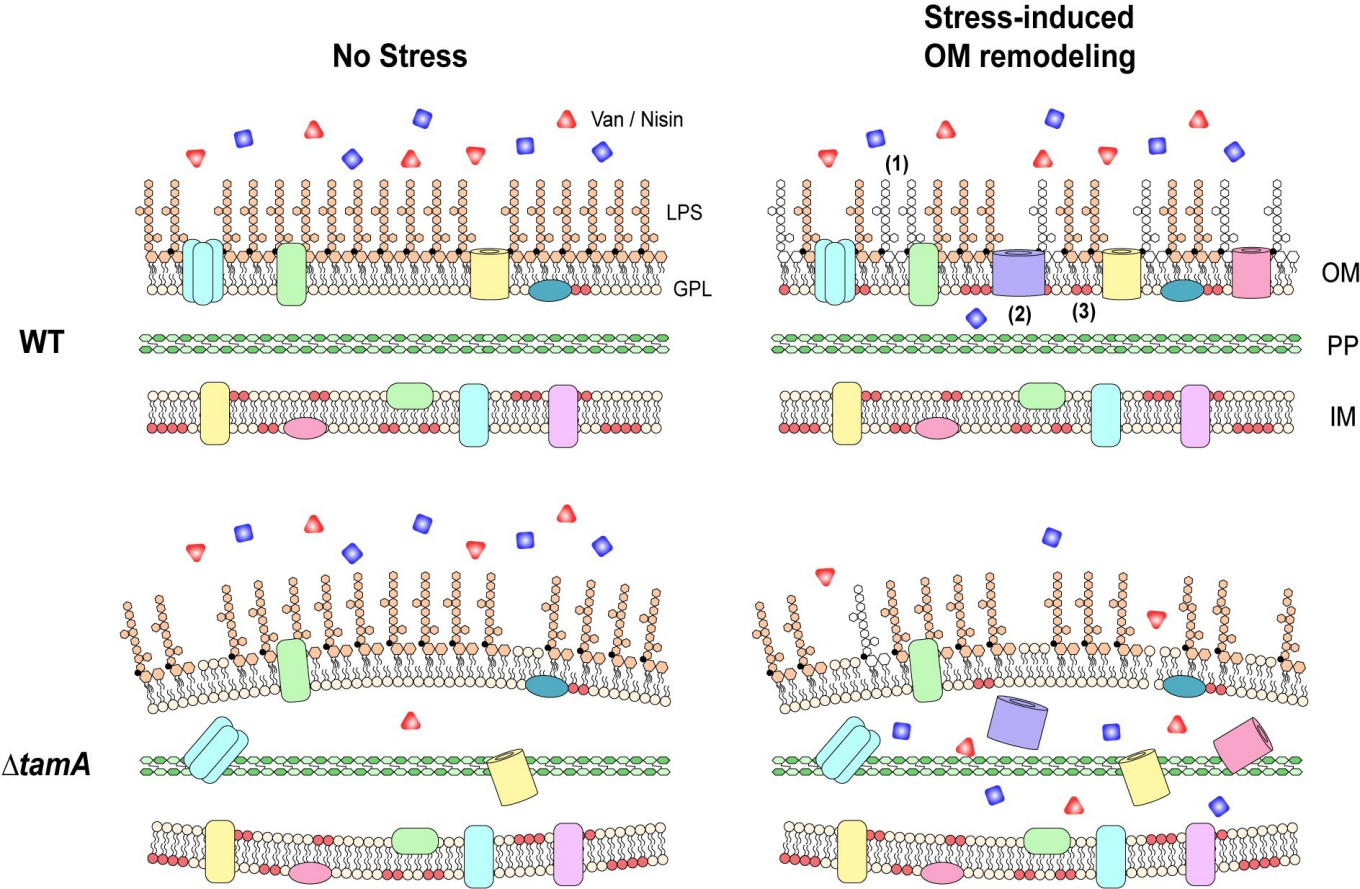
TAM mediates stress-induced OM remodeling, maintaining resistance of CR*-Kp* to antimicrobials in the host. A working model for the role of TAM in antibiotic and antimicrobial peptide resistance during host infection. As a stress response in the host, Gram-negative pathogens, including CR-*Kp*, remodel their outer membrane to maintain its integrity as an effective barrier. This involves modification of (1) LPS, (2) outer membrane proteins, and (3) lipid compositions. When stress-induced OM remodeling is impaired, as observed in Δ*tamA*, OM permeability is increased, leading to higher susceptibility to multiple antimicrobials and thus enhanced clearance of CR-*Kp* from sites of infection.

Under stress conditions, Gram-negative pathogens often modify their outer membrane to promote their survival [40,42,54] (Fig 6). For example, in response to antibiotic stress, the PhoP/PhoQ system of *Salmonella* alters not only LPS and OM proteins but also the glycerophospholipid content of membranes [9,55]—thereby enhancing the barrier function of the OM and increasing resistance to cationic antimicrobial peptides. The increased sensitivity of Δ*tamA* under stress conditions suggests that TAM might contribute to stress-induced remodeling of the OM. Based on the suggested function of TAM in assembly of outer membrane β-barrel proteins [16], we assessed protein profiles of IM/OM/PP fractions and showed that the abundance of several proteins in relation to stress responses or antibiotic resistance were reduced in Δ*tamA*. For example, *gltI* and *djlA* mutants are sensitive to osmotic stresses [56,57] while *sitA*, *hemH*, *asnB*, *djlA* are associated with oxidative stress responses [57–60]; *purM*, *asnB*, *hemL* mutants have increased susceptibility to antibiotics [61–63]—the levels of all these proteins were reduced in Δ*tamA*. A recent study also showed that NlpD, the presence of which was decreased in the IM fraction but increased in the PP fraction of Δ*tamA*, participates in the cell wall remodeling and OM invagination during cytokinesis [64]. While the impact of the individual changes can be small, the alteration of multiple stress-related proteins can exert significant effects collectively on adaptive OM remodeling of Δ*tamA*. Of note, the protein samples in this study were prepared from cells cultured in regular M9 minimal media for effective stable-isotope labeling. It is possible that protein profiles diverge more dramatically if cells are cultured under stress conditions rather than in regular, non-stress-inducing media. While proteomic analysis of stress-induced changes would require careful and extensive studies, SDS-PAGE of the OM fractions from wild type and Δ*tamA* revealed a group of proteins whose abundance differ under stress condition, supporting the possibility that Δ*tamA* is defective in stress-induced OM remodeling (S8A Fig). Meanwhile, another group of proteins showed differential abundance in wild type and Δ*tamA* even in non-stress conditions, and these proteins may also contribute to the differential stress response.

Another possibility to explain the Δ*tamA* phenotype is defective transport of phospholipids under stress conditions. Δ*tamA* showed selective susceptibility to triton X-100, but not to SDS. In contrast to SDS, Triton X-100 is a milder non-ionic detergent and membrane solubilization is dependent on lipid composition [65]. It is possible that the lipid composition of the OM differs between wild type and Δ*tamA*, leading to the selective susceptibility to triton X-100. In line with this idea, Δ*tamA* only demonstrated higher sensitivity to LCA, the most hydrophobic bile acid [66], but not to other bile acids. How can the OM lipid composition be altered in Δ*tamA*? TAM might mediate the assembly of outer membrane proteins involved in lipid transport, such as the Mla system [10–12]. While not previously explored, it is also possible that TAM has a more direct role in lipid transport between the IM and OM—a previous *in silico* study identified TamB, an inner component of the TAM, as a bacterial homologues of eukaryotic lipid transfer proteins [67]. Further studies will be required to explore this possibility.

Previous studies showed that when LPS or resistance-nodulation-division (RND) pumps are impaired, large OM-impermeable antibiotics such as vancomycin can be potent against Gram-negative bacteria [27,68–70]. As mentioned above, they are components of the OM that are often altered by stress [54,55]. As judged by SDS-PAGE and silver staining, we observed some differences in LPS mobility of wild type and Δ*tamA*, particularly when the cells were cultured in low-salt media—which suggests that changes in LPS might contribute to the stress-induced Δ*tamA* phenotype. It also remains possible that delicate changes in lipid A of LPS, not detectible by SDS-PAGE, contribute to the stress-induced Δ*tamA* phenotype. On the other hand, the influx of NPN in Δ*tamA* under hypo-osmotic stress was largely decreased in the absence of CCCP, implying NPN was effectively pumped out in Δ*tamA* [71]. In line with this, the levels of some efflux pumps were increased in Δ*tamA* (Figs 3 and S7), perhaps as a compensatory response to the increased OM permeability.

Because protein sub-fractionation is imperfect, with unavoidable contamination by abundant proteins, extra caution must be taken when concluding about the localization of proteins in each sub-fraction. Initially, we compared two different fractionation methods that were previously established with the closely related gammaproteobacteria, *E*. *coli* [33]. We obtained discrete IM and OM fractions with selective detergents, but not with sucrose density gradient centrifugation, even with different gradient ranges (S6A Fig). Therefore, the detergent method was adopted in this study to separate the IM and OM fractions. To determine effective sub-fractionation, we *in silico* predicted IM, OM, PP proteins from the MH258 genome by PSORTb v3.0 [72] and examined their localization in wild type samples (S6B–S6D Fig). Many of the OM and PP proteins were enriched in the OM and PP fractions, respectively, whereas enrichment was not robust for the IM proteins, suggesting that characteristics of the IM/OM membranes and associated proteins can differ even among closely related bacterial species/strains and that 2% Triton X-100 to elute IM proteins from IM+OM mixture was suboptimal for MH258 strain. Proteins may localize similarly but behave differently depending on their structure, biochemical properties, and surrounding microenvironments. In addition, if the property of the OM membrane is altered in Δ*tamA*, as discussed above, it can also affect selective solubilization of membrane proteins by detergents. On the other hand, the PP fraction was isolated by spheroblasting, making it less likely to be affected by differential membrane properties of wild type and Δ*tamA*.

A recent study showed that gammaproteobacteria increased in abundance during osmotic perturbation in the gut [44]—suggesting that these bacteria might be more resistant to osmotic stress. Combined with multidrug resistance, treatment of such pathogens, including CR-*Kp*, with antibiotics remains an important clinical challenge. In that respect, the potential role of TAM in stress-induces remodeling of the OM might provide novel therapeutic opportunities to develop antibiotic adjuvants that potentiate otherwise ineffective antibiotics. Similar to BamA, one of the targets previously explored to inhibit the OM biogenesis [8], TamA is exposed on the surface, bypassing resistance to inhibitors that is mediated by MDR efflux pumps [73]. In addition, the loss of TAM minimally affects the survival or growth of CR-*Kp*, thus lowering selective pressure for resistance [74]. The current finding might offer an exploitable target for therapeutic agents that enhance the permeability of the outer membrane of Gram-negative bacteria and increase susceptibility to a range of existing and potential antimicrobial agents.

## Materials and methods

### Ethics statement

All mouse experiments were performed in accordance with and approved by the Institutional Animal Care and Use Committee of the University of Chicago under protocol 72599(1).

### Bacterial strains and growth conditions

MH258 is a ST258 CR-*Kp* isolate from a bacteremia patient at MSKCC [75] and all the mutants described in this study were generated on that strain [15]. Unless otherwise stated, all the bacteria were grown in Luria-Bertani (LB) broth or on LB agar at 37°C. As appropriate, the following antibiotics were added to the media: carbenicillin (100 μg/ml), neomycin (50 μg/ml), streptomycin (50 μg/ml), and rifampicin (25 μg/ml).

### *Ex vivo* cecal content cultures

Naïve (previously not exposed to antibiotics) or antibiotic-treated (vancomycin and metronidazole in drinking water for a week) mice were euthanized and the cecal contents were collected in water, PBS or solutions of interest at 100 mg/ml. The suspension was first centrifuged at 3,500 g for 10 min and serially filtered through 0.45-μm and 0.22-μm filters. For the study in S1B Fig, the cecal suspension was incubated for 24 h in an anaerobic chamber (Coy Laboratory Products) with 2.8–4.0% hydrogen before filtration. In some experiments, the filtrates were autoclaved through a liquid cycle for 15 min. The inoculum was prepared by diluting a fresh culture of bacteria at late exponential phase (OD_600_ = 0.8–1.0) with PBS and 20 μl of the inoculum (~ 10^4^ CFU) was added to 180 μl of the cecal filtrates on a 96-well plate. For the competitive study, wild type and each mutant strains were mixed at 1:1 (~5 x 10^3^ CFU of each strain) for inoculation. The plate was incubated at 37°C and the bacterial growth was monitored by track-plating serial dilutions of the cultures on LB agar plates without or with rifampicin (in addition to carbenicillin and neomycin).

### *In vitro* stress responses

A fresh culture of bacteria at late exponential phase (OD_600_ = 0.8–1.0) was washed twice and diluted at 1:1000 (Fig 2A–2F) or 1:10 (S3 and S4 Figs) with PBS. 20 μl of the dilution (~ 10^4^ CFU for Fig 2A–2F; ~ 10^6^ CFU for S3 and S4 Figs) was added to 180 μl of regular LB media, low-salt LB media, PBS or 1/4-diluted PBS with Triton X-100 (0.1% or 1%), SDS (0.01%, 0.1%, or 1%), H_2_O_2_ (2 mM or 5 mM), polymyxin B (2 mM, 10 mM, or 50 mM), 250 uM bile acids (TCA, CA, CDCA, DCA, or LCA), vancomycin (0.1 mg/ml or 1 mg/ml), metronidazole (0.1 mg/ml or 1 mg/ml), carbenicillin (0.1 mg/ml or 1 mg/ml), neomycin (0.05 mg/ml or 0.5 mg/ml), or nisin (2.0 mg/ml; ~15uM). After 1, 3, 6 h incubation at 37°C, the CFU of each culture was determined by plating serial dilutions. Nisin stock was prepared in 0.05% acetic acid; bile acid stocks were prepared in DMSO—equivalent amounts of 0.05% acetic acid or DMSO were used as untreated controls. Low-salt LB media (with 34mM NaCl; 0.2X) was prepared by combining regular LB media (10 g tryptone, 5 g yeast extract, 10 g NaCl in 1L) and no-salt LB media (10 g tryptone, 5 g yeast extract in 1L) at 1:4; 1/4-diluted PBS was prepared by diluting 1X PBS with water. For the dot plating experiment in Figs 2G and S5, either a fresh exponential phase culture or an overnight stationary phase culture was serially diluted and 5 ul of each dilution was blotted on LB plates with differential concentrations of NaCl or EDTA and 0, 0.1, 0.5 mg/ml of vancomycin. The images were taken using the iBright imaging system (Invitrogen).

### NPN uptake study

A fresh culture of bacteria at mid exponential phase (OD_600_ ≈ 0.5) was washed twice with 5 mM isosmotic HEPES buffer (pH 7.2; 137mM NaCl), and then suspended with 5 mM HEPES buffers with 137 mM (1X) or 34.25 mM (0.25X) NaCl. After 10 min incubation with 10 uM CCCP, 100 ul of the cell suspension was mixed with 100ul of 10 uM NPN and the fluorescence signal (excitation, 355/20nm; emission, 405/20nm) was measured within 2 min on the Cytation 5 plate reader (Biotek)—the fluorescence signal was recorded every 1 min for 30 min. The NPN stock (20 mM) was prepared in acetone and diluted with the cell suspension HEPES buffers (either 1X or 0.25X) before use; the CCCP stock (10 mM) was prepared in ethanol. The entire sample preparation processes were performed at room temperature (RT).

### Proteomic analysis

Bacteria colonies on a freshly streaked LB agar plate were inoculated in M9 minimal media supplemented with 0.5% glucose, 1 mM MgSO_4_, 10 uM CaCl_2_, and 0.025% lysine (Lys0 for wild type; Lys8 for Δ*tamA*) and incubated at 37°C until the OD_600_ reached to 0.3–0.5. 5 ml of the cultures were added to a fresh 250 ml M9 media (same to the above) and incubated at 37°C for 3 h. At OD_600_ ≈ 0.5, the cells were harvested and the PP, IM, OM fractions were prepared as described previously [33]. In brief, the cell pellets were suspended in 0.2 M Tris-HCl buffer (pH 8.0) supplemented with 1 M D-sucrose, 1 mM EDTA, 1 mg/ml lysozyme, and a protease inhibitor cocktail (Roche), and the suspension of wild type and Δ*tamA* cells were mixed at 1:1. 4X volume of water was then added to the mixture and incubated for 20 min at RT for spheroblasting. The suspension was centrifuged at 200,000g for 45 min at 4°C and the supernatant was collected as the PP fraction. Next, to separate the membrane faction from the cytoplasmic fraction, the pellet was resuspended in 10 mM Tris-HCl buffer (pH 7.5) supplemented with 5 mM EDTA, 0.2 mM DTT, 6.66 ug/ml DNaseI, and a protease inhibitor cocktail; homogenized by passing through a French Press (Glen Mills) twice at 10^8^ Pa; and spun at 300,000g for 3 h at 4°C. The IM fraction was prepared by suspending the pellet with 50 mM Tris-HCl buffer (pH 8.0) supplemented with 2% Triton X-100 and 10 mM MgCl_2_ and centrifuging at 85,000g for 30 min at 4°C. Lastly, the OM fraction was eluted from the pellet with 4X LDS sample buffer (Invitrogen). The PP and IM fractions were further concentrated using the Amicon centrifugal filter units with 3kDa cut-off before submitting to the Northwestern Proteomics Core Facility for LC-MS/MS. 60 ug of the proteins were gel-purified from each fraction sample and in-gel-digested prior to the mass spectrometry acquisition. The data processing was performed using MaxQuant software [76] to measure the intensity and ratio of heavy and light labels. Potential autotransporters were identified by searching MH258 protein database for proteins similar to known or predicted autotransporters [77] using Blastp with an E-value cut-off of 1.

### Mouse experiments

For intestinal colonization studies, mice were treated with vancomycin (1 g/L), metronidazole (1 g/L), or ampicillin (0.5 g/L) in drinking water for 3 days and inoculated with either a single strain (~1 x 10^5^ CFU) or 1:1 mixture of wild type and each mutant strains (~5 x 10^4^ CFU of each strain) in 200 μl PBS by oral gavage. At the time of inoculation, mice were single-housed and kept on the antibiotics throughout the studies except the FMT experiment in Fig 4F and 4G, in which the antibiotics were lifted upon inoculation. The CBBP bacterial cultures for BCT were prepared by suspending cells—grown individually on Columbia blood agar plates as a lawn for 24 h in an anaerobic chamber—with reduced PBS. Each suspension (~10^8^ CFU/ml) was then mixed at 1:1:1:1 and 200ul of the mixture was administered to mice. The FMTs were prepared by suspending fecal pellets from naïve mice—that were not previously exposed to antibiotics—in reduced PBS (1 pellet per ml), and 200 ul of the suspension was oral-gavaged to each mouse. Both BCT and FMT were administered on 3 consecutive days in a row with freshly prepared cultures. The density of each CR-*Kp* strain in feces and the competitive index (CI, a ratio of mutant CFUs to wild type CFUs normalized to the input ratio) were determined by plating serial dilutions of the fecal samples as described previously [15].

For the lung infection studies, mice were anesthetized with inhaling isoflurane and inoculated intratracheally with ~1 x 10^8^ CFU of each strain in 50 μl PBS. To determine the CFUs, the lung was harvested from the infected mice at the designated time points and the homogenates were plated after serial dilutions. For the bacteremia model, mice were infected with ~1 x 10^7^ CFU of each strain in 100 μl PBS by intraperitoneal injection.

6–8 week-old wild-type C57BL/6 female mice (Jackson Laboratory) were used for all the mouse studies. All mice were maintained under specific pathogen–free conditions at the University of Chicago Animal Resource Center. For the intestinal colonization studies, mice were singly housed upon oral inoculation of CR-*Kp* strains; for infection studies, mice infected with different strains of CR-*Kp* were co-housed. To avoid potential artifacts from variations among cages, individual mice from each cage were randomly assigned to different experimental groups for inoculation or infection.

### Serum killing assay

Bacterial cultures at OD_600_ = 0.8–1.0 were diluted at 1:1000 with PBS and 20 μl of the dilution was mixed with 180 μl of normal human sera (Gemini). The plate was incubated at 37°C for 6 h, and the bacterial survival was monitored by track-plating of serial dilutions. Heat inactivation of the sera was performed by incubating them in a water bath at 56°C for 30 min.

### Statistical analysis

Statistical tests were performed using GraphPad Prism 8. Details of statistical tests and sample sizes are provided in the results and figure legends.

Supplementary methods can be found in S1 Text.

## Supporting information

S1 Fig Cecal contents from naïve mice suppress growth of CR*-Kp* following anaerobic pre-incubation. Growth of wild type and Δ*tamA* strains was compared in cecal filtrates from naïve and antibiotic (ABX)–treated mice (A) without or (B) with 24h incubation of the cecal contents in an anaerobic chamber before filtration. As previously reported [22], growth of both strains was suppressed by antibiotic-naïve cecal contents which had been pre-incubated anaerobically for 24h. The growth inhibition was enhanced when cecal contents were suspended in water; Δ*tamA* grew slightly slower than wild type in this condition. Bar graphs represent geometric means. *ns*, not significant; *ns*, not significant; *, *p* < 0.05; **, *p* < 0.01; ***, *p* < 0.001, by unpaired multiple *t* test on log10 transformation.
(TIF)

S2 Fig Δ*tamA* and wild type bacteria are similar in the use of diverse carbon sources and grow similarly under osmotic and pH stresses. Wild type and Δ*tamA* strains were tested for anaerobic growth on Biolog PM1, 2, 9, and 10 plates over 24 h. On all the tested plates, the growth of wild type and Δ*tamA* strains were comparable as indicated by similar color development over time. Triplicates in two independent experiments were examined and representative images are shown.
(TIF)

S3 Fig Δ*tamA* is more susceptible to triton X-100 and hydrogen peroxide, but not to SDS and polymyxin B under low osmotic stress. Wild type (white circles) and Δ*tamA* (grey circles) strains were compared for their sensitivity to (A, B) triton X-100, SDS, hydrogen peroxide, and polymyxin B in (A) 1X PBS or (B) 0.25X PBS. (C, D) The sensitivity of wild type (white circles) and Δ*tamA* (grey circles) strains to triton X-100 and SDS was monitored for 3h in (C) 1X PBS or (D) 0.25X PBS, and higher % of SDS was tested, compared to (A, B). Error bars represent geometric means ± 95% confidence intervals. *ns*, not significant; *, *p* < 0.05; **, *p* < 0.01; ***, *p* < 0.001, by unpaired multiple *t* test on log10 transformation.
(TIF)

S4 Fig Δ*tamA* is more susceptible to lithocholic acid (LCA) and vancomycin, but not to other bile acids, metronidazole, carbenicillin, and neomycin under low osmotic stress. Wild type (white circles) and Δ*tamA* (grey circles) strains were compared for their sensitivity to (A, B) taurocholic acid (TCA), cholic acid (CA), chenodeoxycholic acid (CDCA), deoxycholic acid (DCA), and LCA; (C, D) vancomycin, metronidazole, carbenicillin, and neomycin in (A, C) 1X PBS or (B, D) 0.25X PBS. Error bars represent geometric means ± 95% confidence intervals. *ns*, not significant; *, *p* < 0.05; **, *p* < 0.01; ***, *p* < 0.001, by unpaired multiple *t* test on log10 transformation.
(TIF)

S5 Fig Δ*tamA* is more susceptible to vancomycin when the outer membrane is destabilized by osmotic stress or LPS release. 10-fold serial dilutions of exponential or stationary phase cultures were blotted on LB plates with different concentrations of NaCl or EDTA and 0, 0.1, or 0.5 mg/ml of vancomycin. P_E, a wild type strain harboring an empty pACYC177_aadA plasmid; Δ*tamA*_E, Δ*tamA* harboring an empty pACYC177_aadA plasmid; Δ*tamA*_C, Δ*tamA* harboring a complementary plasmid, pTam. The images presented in Fig 2G are highlighted in red.
(TIF)

S6 Fig Protein sub-fractionation using selective detergents. (A) SDS-PAGE analysis of IM and OM fractions prepared by either selective detergent method or sucrose density gradient centrifugation [33]. (B–D) Localization of *in silico* predicted IM, OM, PP proteins (PSORTb v3.0) [72] in WT from the SILAC study in Figs 3 and S7. Relative abundance of *in silico* predicted (B) IM, (C) OM, (D) PP proteins in each fraction are plotted for each replicate in a log10 scale. The percentage of *in silico* predicted proteins with the highest abundance in each fraction is indicated—for example, in (C), 75% of *in silico* predicted and MS-detected OM proteins were most abundant in the OM fraction.
(TIF)

S7 Fig Abundance of the majority of the envelope proteins are unchanged in Δ*tamA*. (A) The proteins that are detected for both wild type and Δ*tamA* only in one replicate—so they could not be included in Fig 3A—are plotted in a log2 scale with the same color scheme to Fig 3A. (B) The list of the proteins that showed more than 2-fold changes in (A).
(TIF)

S8 Fig Osmotic stress–induced alterations in OM proteins and LPS in Δ*tamA*. (A) The OM fractions from wild type and Δ*tamA*, cultured in regular (171mM NaCl) or low-salt (34mM NaCl) LB media, were analyzed by SDS-PAGE using 4–12% and 12% Bis-Tris acrylamide gels. The OM fractions were prepared by chaotropic reagent method [33] to reduce sample handling variations from multiple sub-fractionation steps. Three groups of proteins were observed: (1) proteins whose abundance change in both WT and KO upon osmotic stress (black arrows), (2) proteins whose abundance differ in WT vs. KO even in normal condition (blue arrows), (3) proteins whose abundance differ in WT vs. KO under stress condition (red arrows). (B) A wild type strain harboring an empty pACYC177_aadA plasmid (P_E) and Δ*tamA* harboring either an empty pACYC177_aadA plasmid (Δ*tamA*_E) or a complementary plasmid, pTam, (Δ*tamA*_C) were cultured either in regular (171mM NaCl, 1X) or low-salt (34mM NaCl, 0.25X) LB media to the exponential phase (OD ≈ 0.8), and then crude extracts of LPS were extracted and analyzed on SDS-PAGE followed by silver staining. Only Δ*tamA* from low-salt media showed an alteration (asterisks).
(TIF)

S9 Fig *tamA* and *tamB* gene expression are largely unaltered under osmotic stress. Wild type and Δ*tamA* strains were cultured either in regular (171mM NaCl) or low-salt (34mM NaCl) LB media to the exponential phase (OD ≈ 0.8) and gene expression of *tamA* and *tamB* were analyzed by qRT-PCR. Data were normalized to the levels of *rpoD* and then compared to wild type cultured in regular media. Bar graphs represent means. *ns*, not significant; *, *p* < 0.05; **, *p* < 0.01; ***, *p* < 0.001, by unpaired multiple *t* test.
(TIF)

S10 Fig Comparison of Δ*tamA* and acapsular mutants in virulence. (A) Mice were inoculated intraperitoneally with 10^8^ CFU of wild type, Δ*tamA*, Δ*wza* or Δ*wzc* strains and survival was monitored over 7 days (*n* = 8). Compared to Fig 5C, higher inoculum (10^8^ *vs*. 10^7^ CFU) was used to distinguish Δ*tamA* and acapsular mutants (Δ*wza* and Δ*wzc*) [39]. *ns*, not significant; *, *p* < 0.05; **, *p* < 0.01; ***, *p* < 0.001, by Gehan-Breslow-Wilcoxon test. (B) Wild type, Δ*tamA*, Δ*wza* and Δ*wzc* strains were mono-cultured in normal human sera without or with heat inactivation. Bar graphs represent geometric means. *ns*, not significant; *, *p* < 0.05; **, *p* < 0.01; ***, *p* < 0.001, by unpaired multiple *t* test on log10 transformation.
(TIF)

S1 Table Fold changes and *p*-values of all the proteins plotted in Fig 3A. (XLSX)

S2 Table Intensities of heavy (Δ*tamA*) and light (wild type) labels for the proteins listed in Fig 3B. (XLSX)

S3 Table The levels of predicted β-barrel proteins and autotransporters are minimally altered in Δ*tamA*. (XLSX)

S1 Text Supplementary methods. (DOCX)

S1 Data Raw data of the LC-MS/MS analysis in Figs 3 and S7. (XLSX)
