# Supplementary figures and images for: TAM mediates adaptation of carbapenem-resistant Klebsiella pneumoniae to antimicrobial stress during host colonization and infection
Source: PLoS Pathog. 2021 Feb 8;17(2):e1009309. doi: 10.1371/journal.ppat.1009309 (PMC7895364; doi:10.1371/journal.ppat.1009309)

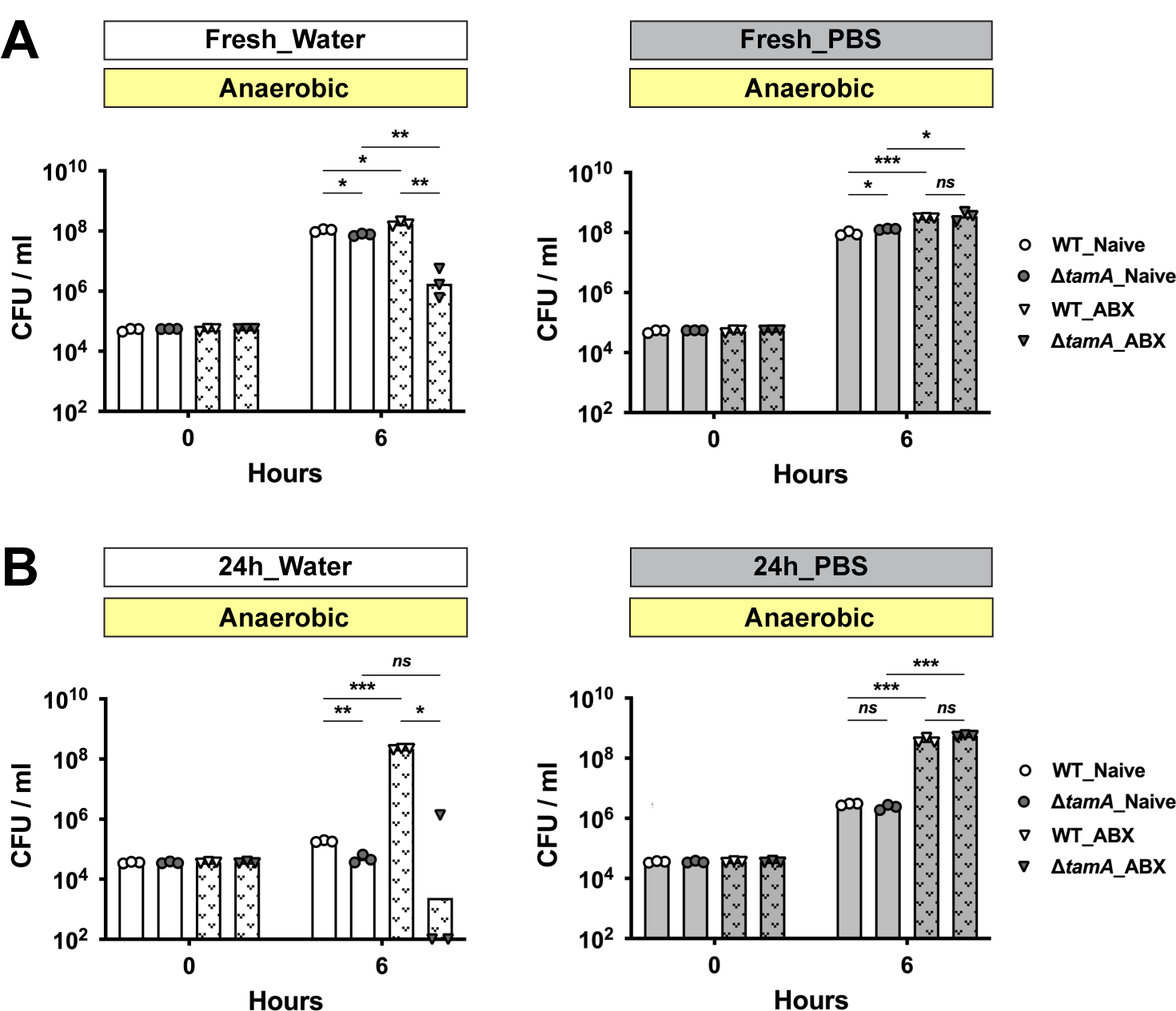

Supplement: S1 Fig — Growth of wild type and ΔtamA strains was compared in cecal filtrates from naïve and antibiotic (ABX)–treated mice (A) without or (B) with 24h incubation of the cecal contents in an anaerobic chamber before filtration. As previously reported [22], growth of both strains was suppressed by antibiotic-naïve cecal contents which had been pre-incubated anaerobically for 24h. The growth inhibition was enhanced when cecal contents were suspended in water; ΔtamA grew slightly slower than wild type in this condition. Bar graphs represent geometric means. ns, not significant; ns, not significant; *, p < 0.05; **, p < 0.01; ***, p < 0.001, by unpaired multiple t test on log10 transformation. (TIF) [file ppat.1009309.s001.tif]

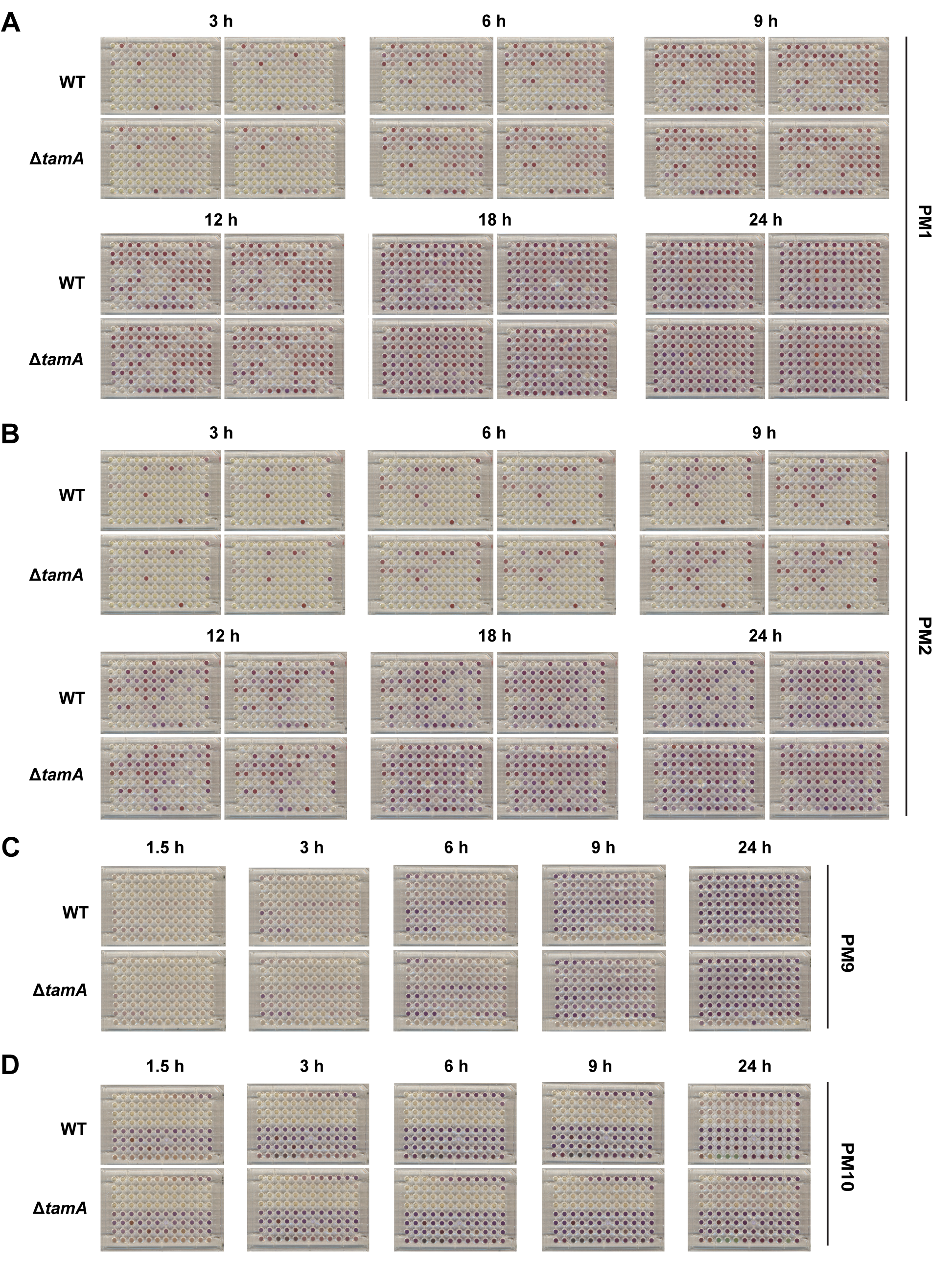

Supplement: S2 Fig — Wild type and ΔtamA strains were tested for anaerobic growth on Biolog PM1, 2, 9, and 10 plates over 24 h. On all the tested plates, the growth of wild type and ΔtamA strains were comparable as indicated by similar color development over time. Triplicates in two independent experiments were examined and representative images are shown. (TIF) [file ppat.1009309.s002.tif]

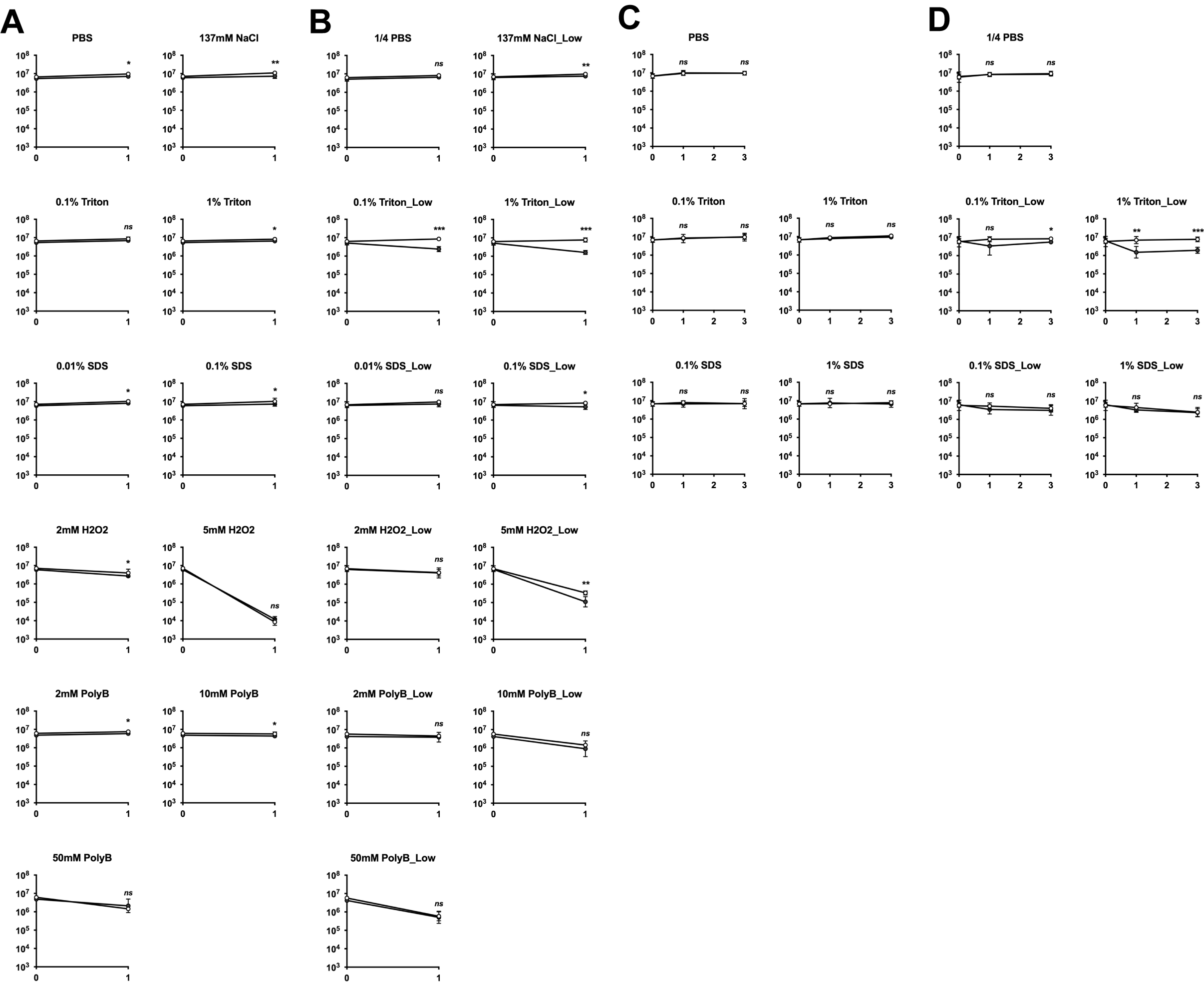

Supplement: S3 Fig — Wild type (white circles) and ΔtamA (grey circles) strains were compared for their sensitivity to (A, B) triton X-100, SDS, hydrogen peroxide, and polymyxin B in (A) 1X PBS or (B) 0.25X PBS. (C, D) The sensitivity of wild type (white circles) and ΔtamA (grey circles) strains to triton X-100 and SDS was monitored for 3h in (C) 1X PBS or (D) 0.25X PBS, and higher % of SDS was tested, compared to (A, B). Error bars represent geometric means ± 95% confidence intervals. ns, not significant; *, p < 0.05; **, p < 0.01; ***, p < 0.001, by unpaired multiple t test on log10 transformation. (TIF) [file ppat.1009309.s003.tif]

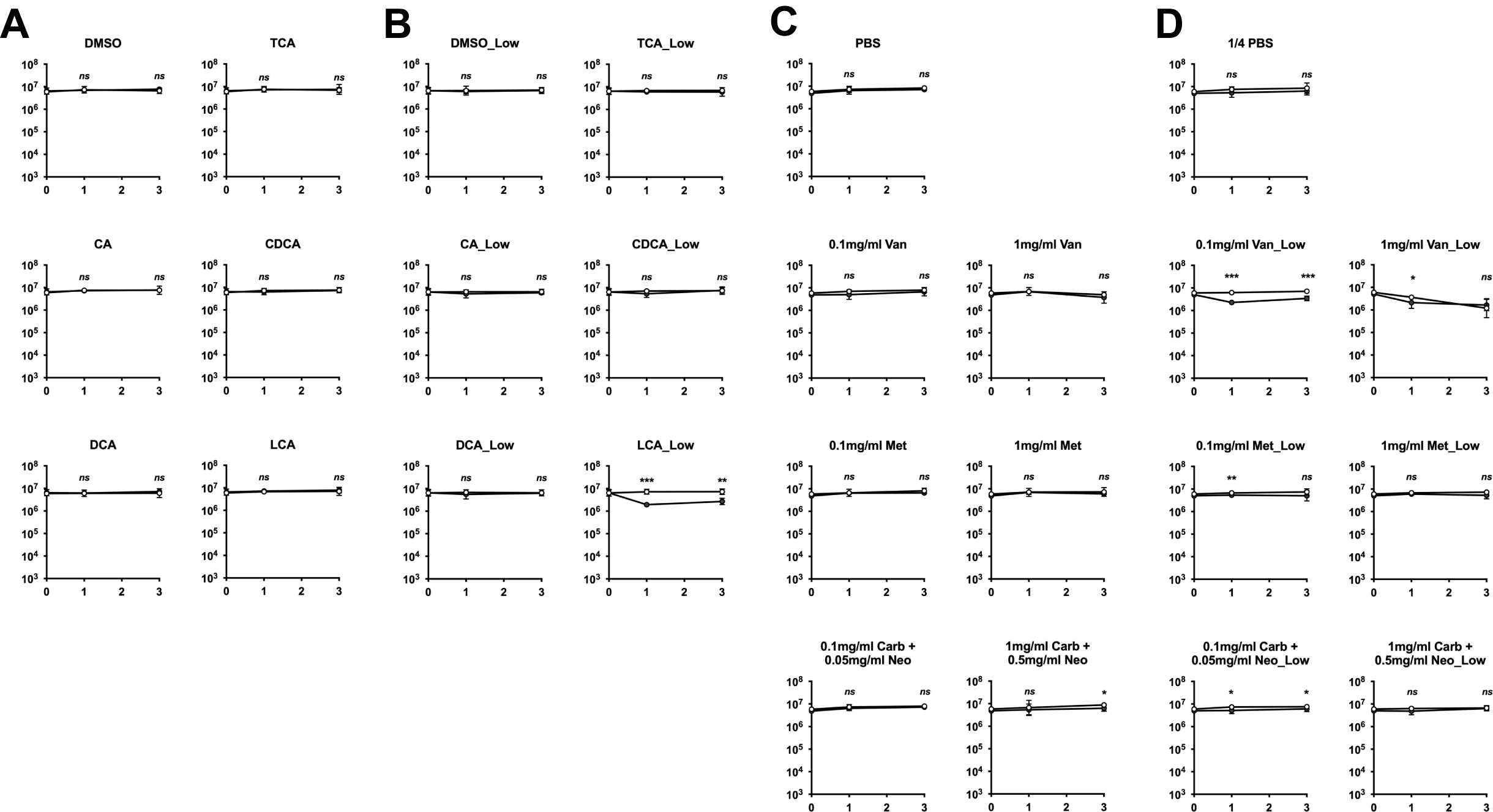

Supplement: S4 Fig — Wild type (white circles) and ΔtamA (grey circles) strains were compared for their sensitivity to (A, B) taurocholic acid (TCA), cholic acid (CA), chenodeoxycholic acid (CDCA), deoxycholic acid (DCA), and LCA; (C, D) vancomycin, metronidazole, carbenicillin, and neomycin in (A, C) 1X PBS or (B, D) 0.25X PBS. Error bars represent geometric means ± 95% confidence intervals. ns, not significant; *, p < 0.05; **, p < 0.01; ***, p < 0.001, by unpaired multiple t test on log10 transformation. (TIF) [file ppat.1009309.s004.tif]

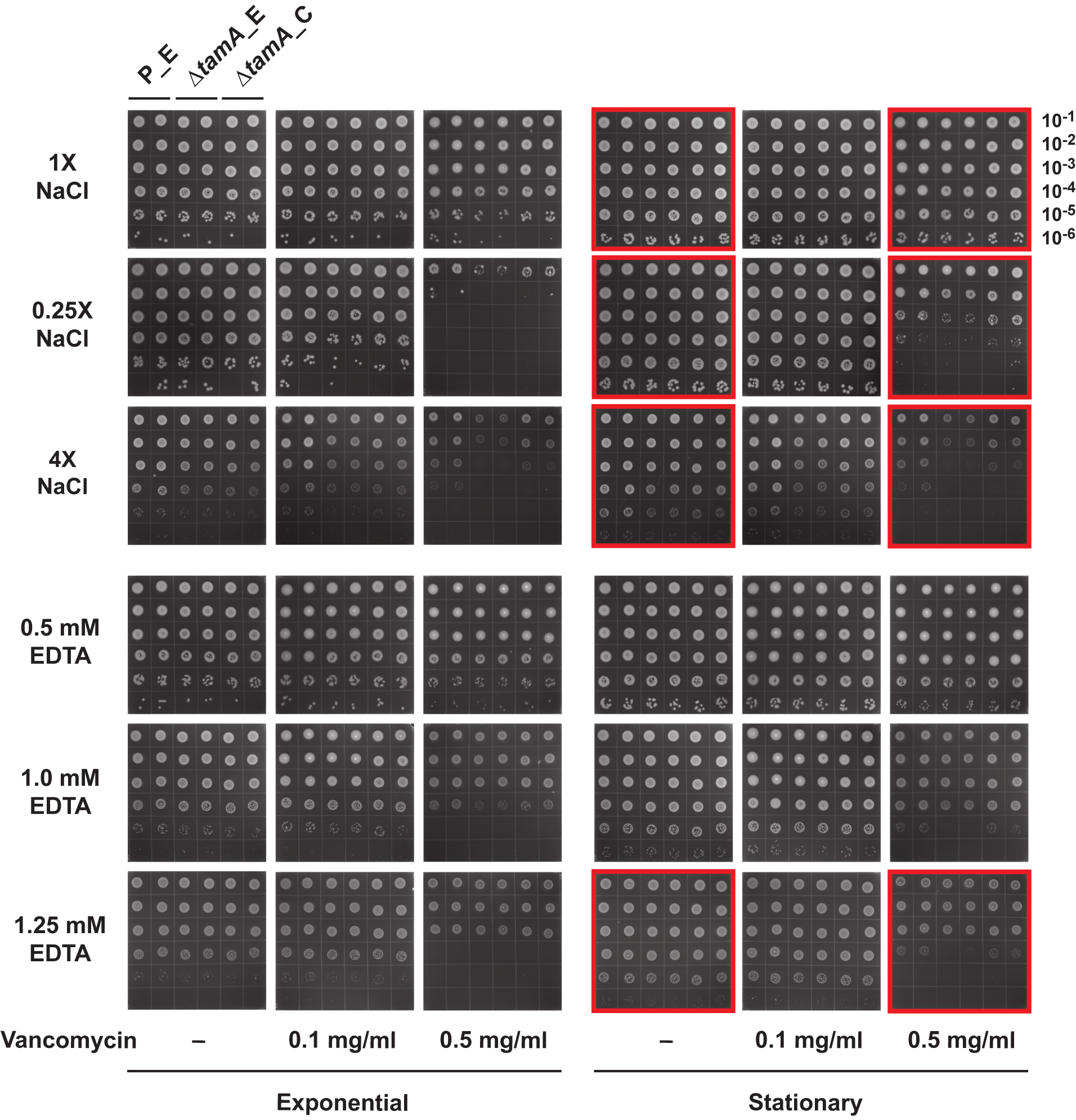

Supplement: S5 Fig — 10-fold serial dilutions of exponential or stationary phase cultures were blotted on LB plates with different concentrations of NaCl or EDTA and 0, 0.1, or 0.5 mg/ml of vancomycin. P_E, a wild type strain harboring an empty pACYC177_aadA plasmid; ΔtamA_E, ΔtamA harboring an empty pACYC177_aadA plasmid; ΔtamA_C, ΔtamA harboring a complementary plasmid, pTam. The images presented in Fig 2G are highlighted in red. (TIF) [file ppat.1009309.s005.tif]

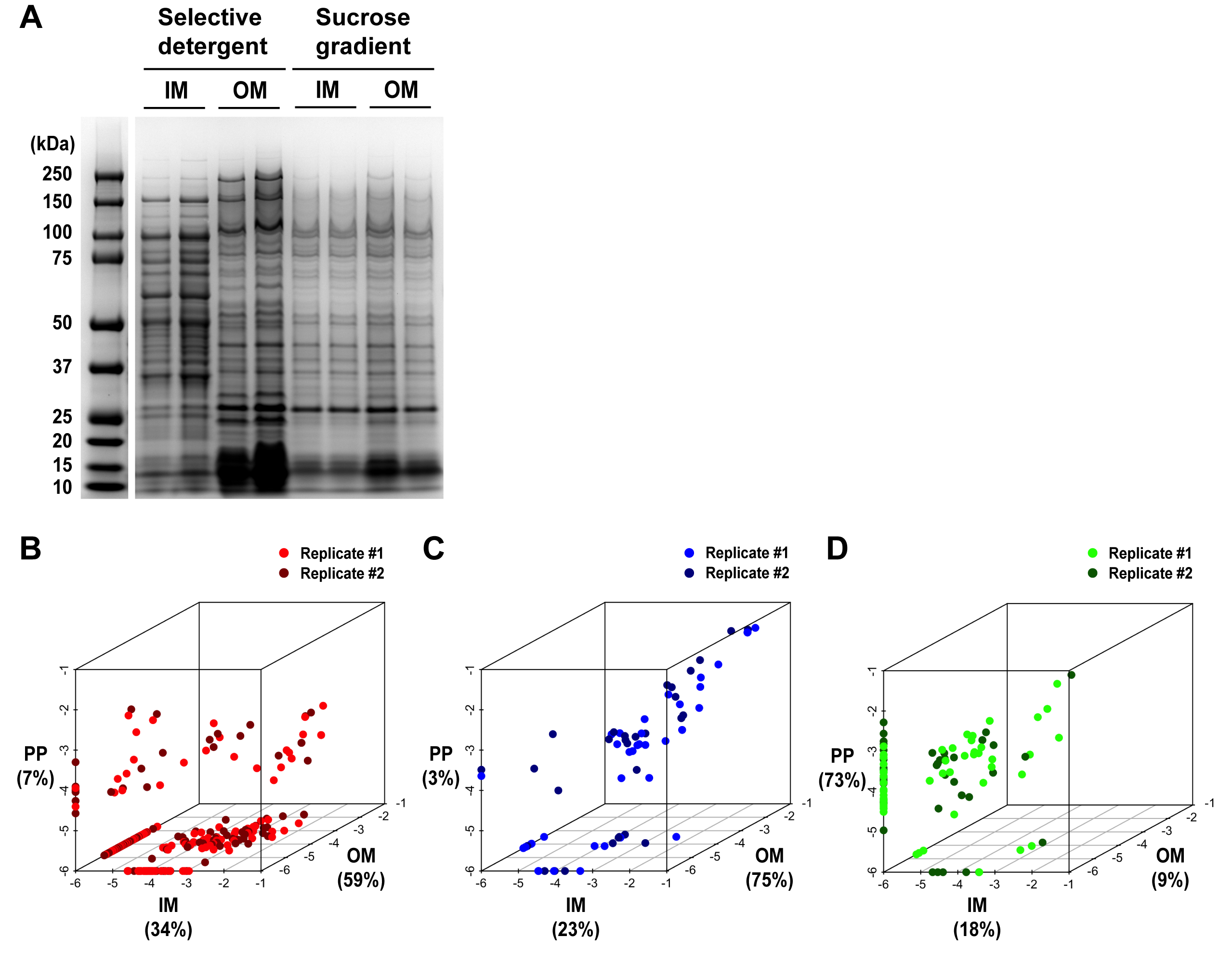

Supplement: S6 Fig — (A) SDS-PAGE analysis of IM and OM fractions prepared by either selective detergent method or sucrose density gradient centrifugation [33]. (B–D) Localization of in silico predicted IM, OM, PP proteins (PSORTb v3.0) [72] in WT from the SILAC study in Figs 3 and S7. Relative abundance of in silico predicted (B) IM, (C) OM, (D) PP proteins in each fraction are plotted for each replicate in a log10 scale. The percentage of in silico predicted proteins with the highest abundance in each fraction is indicated—for example, in (C), 75% of in silico predicted and MS-detected OM proteins were most abundant in the OM fraction. (TIF) [file ppat.1009309.s006.tif]

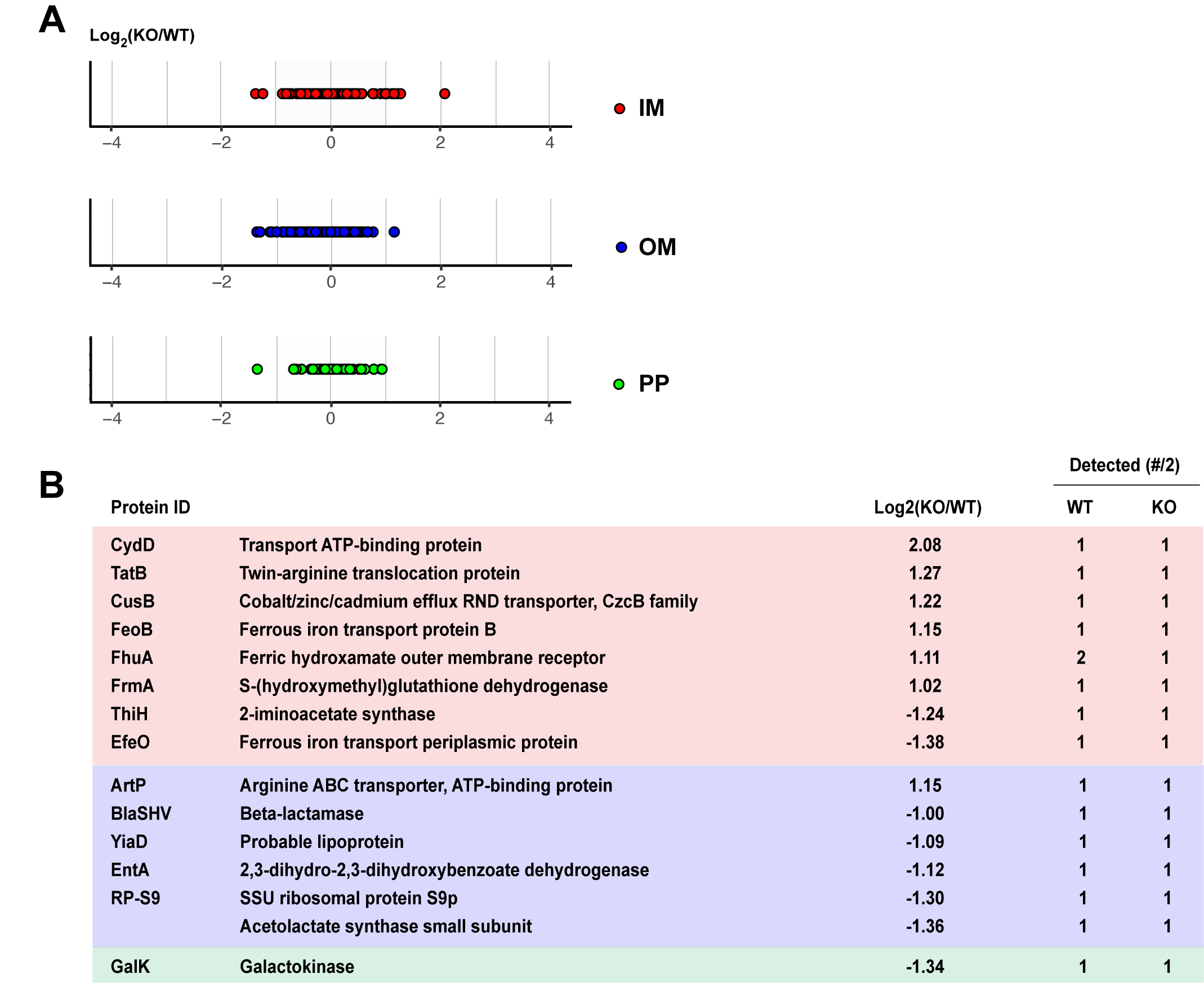

Supplement: S7 Fig — (A) The proteins that are detected for both wild type and ΔtamA only in one replicate—so they could not be included in Fig 3A—are plotted in a log2 scale with the same color scheme to Fig 3A. (B) The list of the proteins that showed more than 2-fold changes in (A). (TIF) [file ppat.1009309.s007.tif]

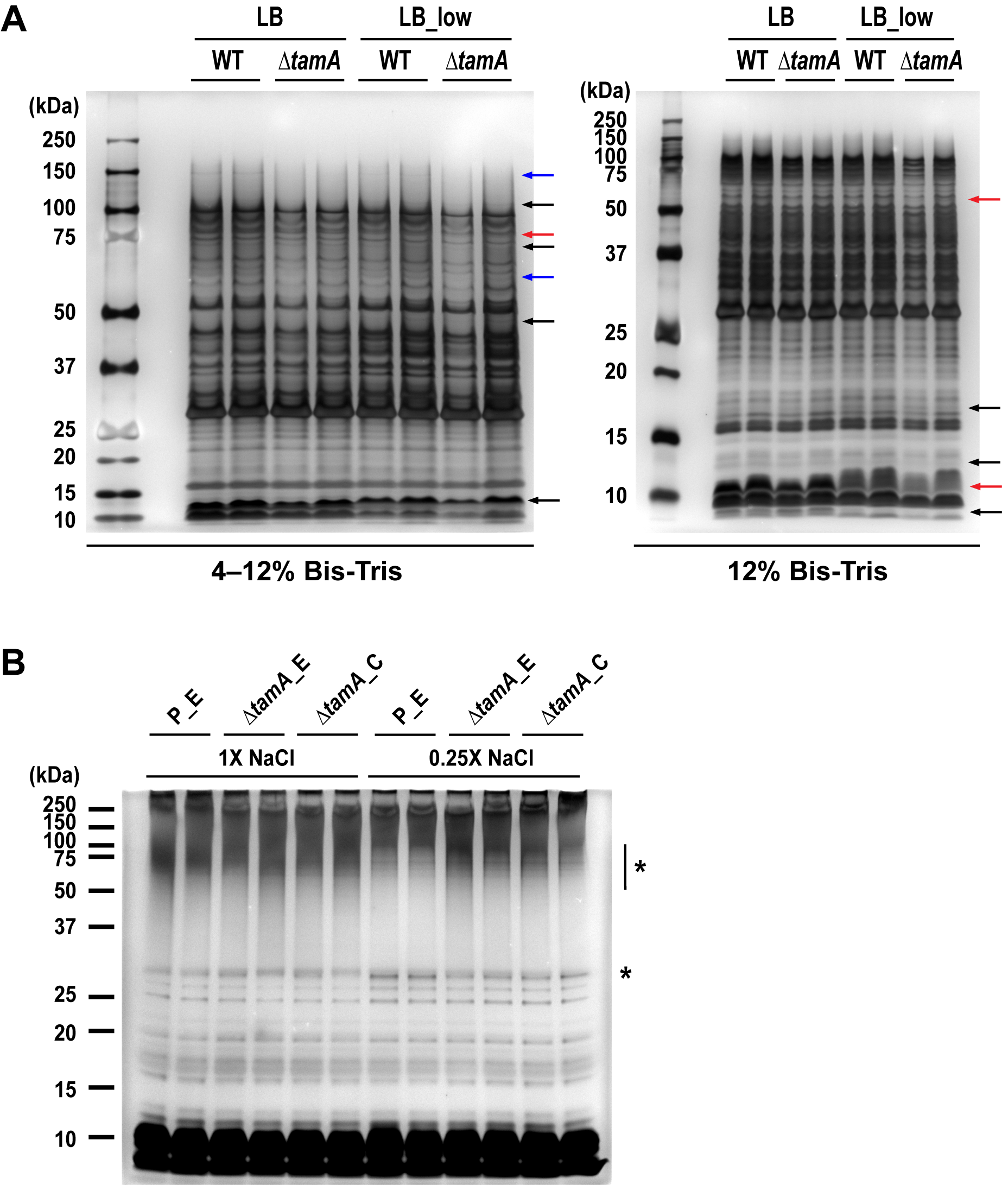

Supplement: S8 Fig — (A) The OM fractions from wild type and ΔtamA, cultured in regular (171mM NaCl) or low-salt (34mM NaCl) LB media, were analyzed by SDS-PAGE using 4–12% and 12% Bis-Tris acrylamide gels. The OM fractions were prepared by chaotropic reagent method [33] to reduce sample handling variations from multiple sub-fractionation steps. Three groups of proteins were observed: (1) proteins whose abundance change in both WT and KO upon osmotic stress (black arrows), (2) proteins whose abundance differ in WT vs. KO even in normal condition (blue arrows), (3) proteins whose abundance differ in WT vs. KO under stress condition (red arrows). (B) A wild type strain harboring an empty pACYC177_aadA plasmid (P_E) and ΔtamA harboring either an empty pACYC177_aadA plasmid (ΔtamA_E) or a complementary plasmid, pTam, (ΔtamA_C) were cultured either in regular (171mM NaCl, 1X) or low-salt (34mM NaCl, 0.25X) LB media to the exponential phase (OD ≈ 0.8), and then crude extracts of LPS were extracted and analyzed on SDS-PAGE followed by silver staining. Only ΔtamA from low-salt media showed an alteration (asterisks). (TIF) [file ppat.1009309.s008.tif]

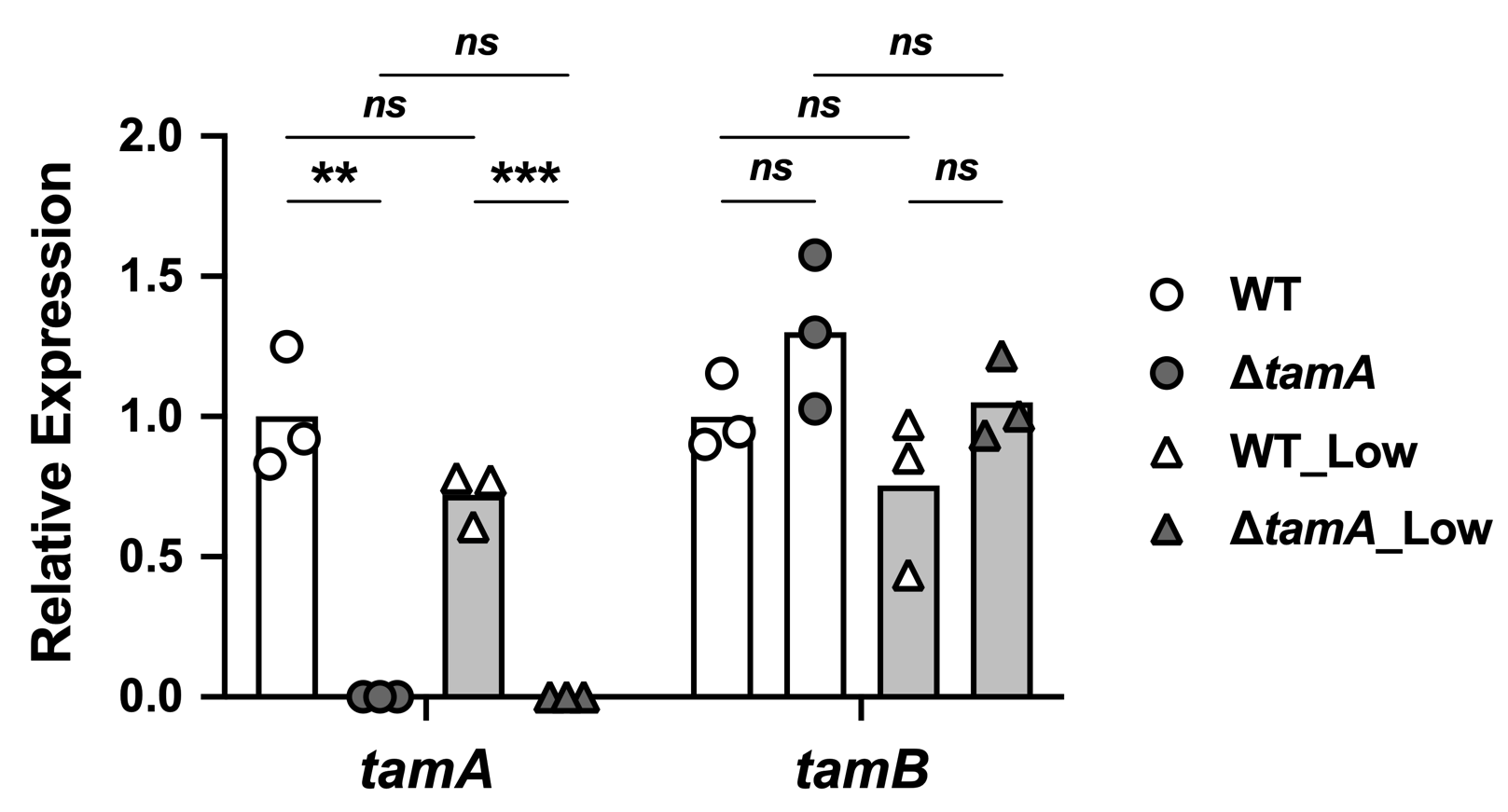

Supplement: S9 Fig — Wild type and ΔtamA strains were cultured either in regular (171mM NaCl) or low-salt (34mM NaCl) LB media to the exponential phase (OD ≈ 0.8) and gene expression of tamA and tamB were analyzed by qRT-PCR. Data were normalized to the levels of rpoD and then compared to wild type cultured in regular media. Bar graphs represent means. ns, not significant; *, p < 0.05; **, p < 0.01; ***, p < 0.001, by unpaired multiple t test. (TIF) [file ppat.1009309.s009.tif]

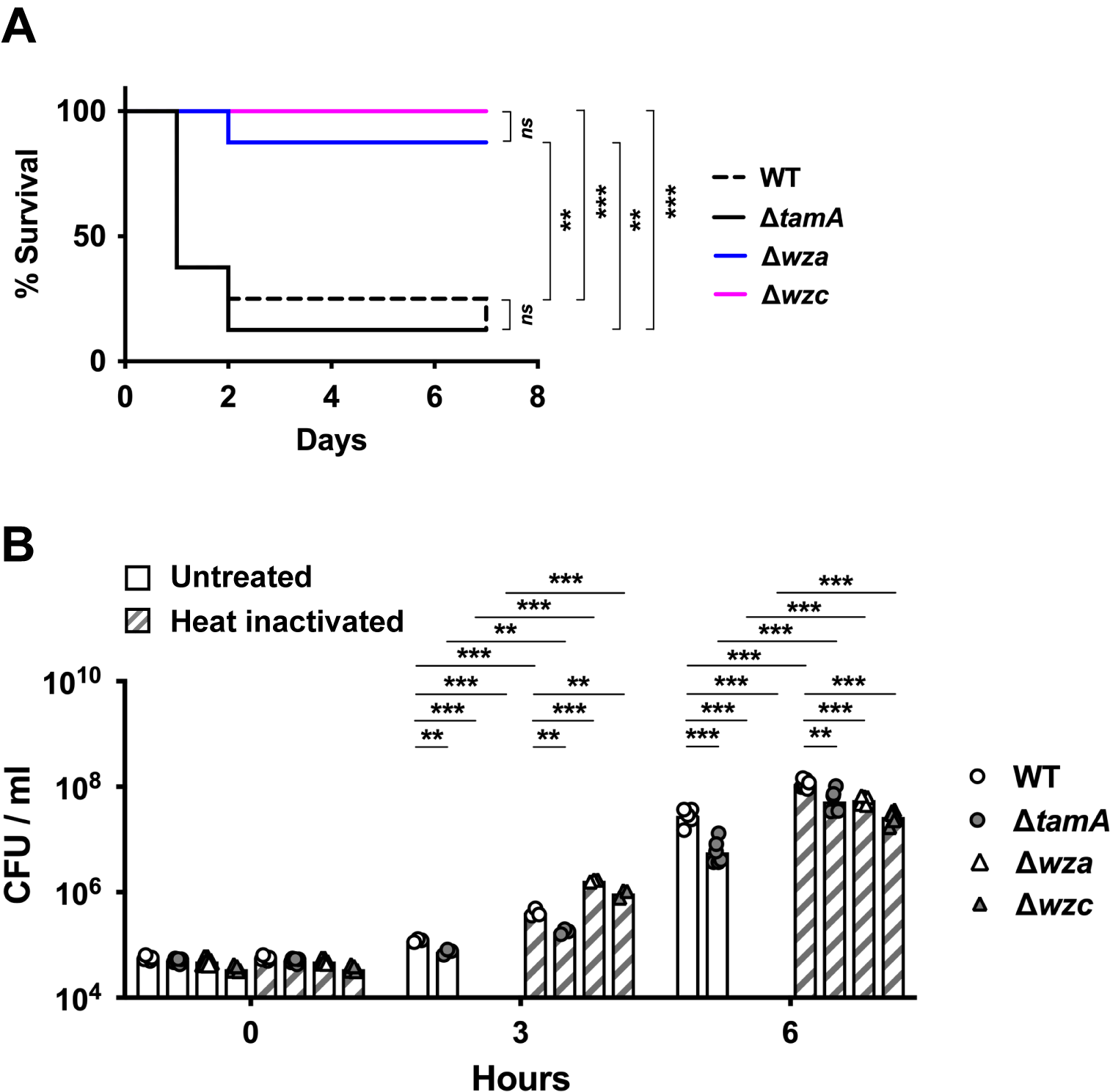

Supplement: S10 Fig — (A) Mice were inoculated intraperitoneally with 108 CFU of wild type, ΔtamA, Δwza or Δwzc strains and survival was monitored over 7 days (n = 8). Compared to Fig 5C, higher inoculum (108 vs. 107 CFU) was used to distinguish ΔtamA and acapsular mutants (Δwza and Δwzc) [39]. ns, not significant; *, p < 0.05; **, p < 0.01; ***, p < 0.001, by Gehan-Breslow-Wilcoxon test. (B) Wild type, ΔtamA, Δwza and Δwzc strains were mono-cultured in normal human sera without or with heat inactivation. Bar graphs represent geometric means. ns, not significant; *, p < 0.05; **, p < 0.01; ***, p < 0.001, by unpaired multiple t test on log10 transformation. (TIF) [file ppat.1009309.s010.tif]
