# Supplementary material for: TAM mediates adaptation of carbapenem-resistant Klebsiella pneumoniae to antimicrobial stress during host colonization and infection
Source: PLoS Pathog. 2021 Feb 8;17(2):e1009309. doi: 10.1371/journal.ppat.1009309 (PMC7895364; doi:10.1371/journal.ppat.1009309)
Supplement: S1 Text — (DOCX) [file ppat.1009309.s014.docx]

**Supplementary methods**

**Biolog experiments**

Bacterial colonies on a freshly streaked plate were inoculated in reduced brain heart infusion (BHI) media and incubated anaerobically at 37°C overnight. Next morning, the cells were harvested and resuspended with reduced IF-0a GN/GP base inoculating fluid (Biolog). The PM inoculating fluids for PM1/2 and 9/10 plates were prepared anaerobically following the manufacturer’s protocol for anaerobic bacteria with dye mix D (Biolog) and mixed with the bacterial suspension at 20:1. The plates were incubated anaerobically at 37°C and their colorimetric changes were monitored over 24 h using a general office scanner.

**Proteomic analysis using chaotropic reagent method**

Bacteria colonies were inoculated in LB media and incubated aerobically at 37°C overnight. Next day, the fresh overnight cultures were inoculated in regular (171mM NaCl) or low-salt (34mM NaCl) LB media at 1:100 and incubated at 37°C until the OD_600_ reached to ~0.8. The cells were then harvested and the OM fractions were prepared as described previously [[1](#_ENREF_1)]. In brief, the cell pellets were suspended in 0.1M Tris-HCl buffer (pH 7.5) supplemented with DNaseI and a protease inhibitor cocktail (Roche), and the suspensions were homogenized by passing through a French Press (Glen Mills) twice at 10^8^ Pa. The homogenates were spun at 4000 rpm for 15 min at 4°C to remove incompletely lysed cells and the supernatants were diluted with 0.1M sodium carbonate at 1:10. After 1 h gentle mixing at 4°C, the suspension was centrifuged at 120,000g for 1 h at 4°C and the pellets were washed twice with water. The OM fractions were eluted from the pellets with 4X LDS sample buffer (Invitrogen) and analyzed on 4–12% and 12% Bis-Tris acrylamide gels (Invitrogen).

**LPS analysis**

Fresh overnight culture of bacteria in LB were inoculated in regular (171mM NaCl) or low-salt (34mM NaCl) LB media at 1:100 and incubated at 37°C. As the OD_600_ reached to ~0.8, the cells were harvested and crude LPS were extracted using LPS extraction kit (iNtRON) following the manufacturer’s protocol. Extracted LPS were treated with proteinase K and analyzed on 12% Bis-Tris acrylamide gels (Invitrogen). LPS were visualized using a silver stain kit (Pierce).

**qRT-PCR**

Bacteria, cultured either in regular (171mM NaCl) or low-salt (34mM NaCl) LB media to the exponential phase (OD ≈ 0.8), were harvested and total RNA were extracted using a TRIzol Max Bacterial RNA Isolation Kit (Invitrogen). cDNAs were synthesized using a QuantiTect Reverse Transcription kit (Qiagen) and qPCR reactions were performed with a PowerUp SYBR Green Master Mix (Applied Biosystems) on the QuantStudio 6 Pro (Applied Biosystems).

**Supplementary reference**

1. Thein M, Sauer G, Paramasivam N, Grin I, Linke D. Efficient subfractionation of gram-negative bacteria for proteomics studies. J Proteome Res. 2010;9(12):6135-47. doi: 10.1021/pr1002438. PubMed PMID: 20932056.
